# Birth and Death Notifications for Improving Civil Registration and Vital Statistics in Bangladesh: Pilot Exploratory Study

**DOI:** 10.2196/25735

**Published:** 2022-08-29

**Authors:** Tazeen Tahsina, Afrin Iqbal, Ahmed Ehsanur Rahman, Suman Kanti Chowdhury, Atique Iqbal Chowdhury, Sk Masum Billah, Ataur Rahman, Monira Parveen, Lubana Ahmed, Qazi Sadequr Rahman, Shah Ali Akbar Ashrafi, Shams El Arifeen

**Affiliations:** 1 International Centre for Diarrhoeal Disease Research, Bangladesh Dhaka Bangladesh; 2 School of Health and Related Research University of Sheffield Sheffield United Kingdom; 3 UNICEF Dhaka Bangladesh; 4 Cowatersogema Dhaka Bangladesh; 5 Civil Registration and Vital Statistics Dhaka Bangladesh

**Keywords:** notification, registration, birth, death, CRVS, mobile phone, mobile app, mobile technology, technology-based platform, community health, low- and middle-income countries, mHealth, Bangladesh

## Abstract

**Background:**

Effective health policy formulation requires sound information of the numerical data and causes of deaths in a population. Currently, in Bangladesh, neither births nor deaths are fully and promptly registered. Birth registration in Bangladesh is around 54% nationally. Although the legal requirements are to register within 45 days of an event, only 4.5% of births and 35.9% of deaths were reported within the required time frame in 2020. This study adopted an innovative digital notification approach to improve the coverage of registration of these events at the community level.

**Objective:**

Our primary objective was to assess (1) the proportion of events identified by the new notification systems (success rate) and the contribution of the different notifiers individually and in combination (completeness) and (2) the proportion of events notified within specific time limits (timeliness of notifications) after introducing the innovative approach.

**Methods:**

We conducted a pilot study in 2016 in 2 subdistricts of Bangladesh to understand whether accurate, timely, and complete information on births and deaths can be collected and notified by facility-based service providers; community health workers, including those who routinely visit households; local government authorities; and key informants from the community. We designed a mobile technology–based platform, an app, and a call center through which the notifications were recorded. All notifications were verified through the confirmation of events by family members during visits to the concerned households. We undertook a household survey–based assessment at the end of the notification period.

**Results:**

Our innovative system gathered 13,377 notifications for births and deaths from all channels, including duplicate reports from multiple sources. Project workers were able to verify 92% of the births and 93% of the deaths through household visits. The household survey conducted among a subsample of the project population identified 1204 births and 341 deaths. After matching the notifications with the household survey, we found that the system was able to capture over 87% of the births in the survey areas. Health assistants and family welfare assistants were the primary sources of information. Notifications from facilities were very low for both events.

**Conclusions:**

The Global Civil Registration and Vital Statistics: Scaling Up Investment Plan 2015-2024 and the World Health Organization reiterated the importance of building an evidence base for improving civil registration and vital statistics. Our pilot innovation revealed that it is possible to coordinate with the routine health information system to note births and deaths as the first step to ensure registration. Health assistants could capture more than half of the notifications as a stand-alone source.

## Introduction

“No one should be without a legal identity. No life should be allowed to remain invisible to policymakers. No person should fall between the cracks of incomplete official data” [1]; this statement stresses the Sustainable Development Goal 16.9 agenda and the recommendations of the United Nations Commission on Information and Accountability for Women and Children's Health, thereby ensuring legal identity for all [2-5]. Births, deaths, marriages, divorce, adoption, and causes of death are the vital events that constitute a country’s civil registration and vital statistics (CRVS). Vital registration is fundamental to the logical operation of health care services, thereby ensuring equity, empowerment, and improved economic productivity [6-11]. Birth registration is an essential human right, acknowledging individual existence and identity. It also helps ensure health care, social protection, and education [9]. Death registration aids the estimation of disease burden and provides an understanding of the cause of death in communities, which is critical for prioritizing strategies and designing interventions addressing specific health needs [12,13].

Birth registration in Bangladesh is around 54% nationally, while death registration only covered 14.5% of deaths [14]. In 2010, the Office of the Registrar General of Birth and Death Registration launched an online registration system, but the coverage has still been low. Out of those registered, only 4.5% of births and 35.9% of deaths were registered within 45 days in 2020 [15,16]. In 2016, the Birth Registration Information System recorded that only 2% of births were registered within 45 days and death registration covered 13% of the national deaths [15]. There are multiple ministries within the government that are involved in the identification of vital events. An interministerial approach labelled as “CRVS++” has been undertaken by the cabinet division since 2014 [17]. The country is still working toward developing a unique ID system and linking it to the service delivery processes of various ministries, including the Ministry of Health and Family Welfare. Efforts are being scaled up to generate the cause of death from health care facilities, and a pilot study was initiated in the Kaliganj subdistrict for community-level workers to conduct verbal autopsies [16,17]. Coverage data on the pilot initiatives are yet to be visible through the CRVS tracker. However, the minimal effort directed toward the identification of cause of death has resulted in large changes in the timely reporting of death events [16].

**Figure 1:**
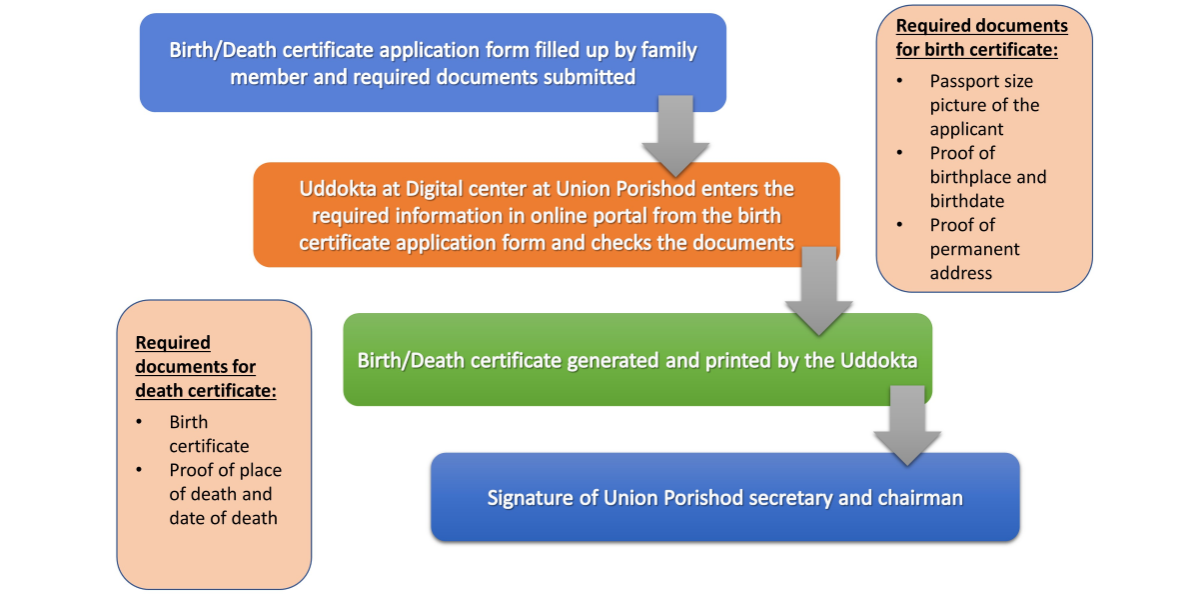
Mechanical process of availing and distributing a legal document for individual record.

In Bangladesh, the birth and death registration process involves 2 steps: (1) identification and notification and (2) registration [18]. The Birth and Death Registration Act 2014 [19] promotes notification within 45 days and identifies a set of entities as possible notifiers across different government bodies and the community [20]. The second part, the registration, is a mechanical process of availing and distributing a legal document, the certificate, for individual records (Figure 1). The notification of births and deaths can be the first step toward increasing the coverage of registration. The health sector can play a pivotal role in improving the notification of births and deaths through innovations [21].

There is a dearth of evidence regarding the feasibility and functionality of using different sources as potential notifiers, particularly the health sector. Every additional source adds an extra level of complexity to the overall system and its accountability and sustainability. The published literature focuses on the need for and importance of a well-functioning CRVS system for countries [6,8,10,13,22-25]. Two country-specific studies and 1 regional study have explained ways to improve the coverage of certificates and how the health system can help improve the CRVS [13,23,26]. However, these tend to be passive notification systems that underperform; none focused on a more proactive and innovative method to improve notification. There is scope for research and innovation to identify an efficient notification process and acknowledge the importance of having an optimum number of notifiers. Once that part is done, the mechanical challenges associated with the registration system can be assessed in the next stage.

Our primary objective was to assess (1) the proportion of events identified by the new notification systems (success rate) and the contribution of different notifiers individually and in combination (completeness) and (2) the proportion of events notified within specific time limits (timeliness of notifications) after introducing the innovative approach. We also aimed to understand whether the system excluded individuals belonging to any specific sociodemographic characteristics or located within specific geographic boundaries from the reporting system.

## Methods

### Description of the Notification Process

Following the Birth and Death Registration Act [19], we identified a list of individuals and authorities eligible to notify an event of birth and death (Table 1). The notifiers, selected in consultation with the Ministry of Health and Family Welfare and local government representatives, fall into 3 groups. These include public facility–level health care service providers (nursing supervisor/nurse and resident medical officer), community-level domiciliary health and family planning workers (health assistants, family welfare assistants, and community health care providers [CHCPs]), and local government representatives, namely, the Uddoktas (a community volunteer who facilitates birth and death registration at the union level), village police, imams (religious leaders), and households. Private sector facilities were not included in the notification process.

**Table 1:** List of channels for the notification of births and deaths in 2 rural subdistricts of Bangladesh from January to October 2016.

Notification interface, institute, work station, and notifiers						Catchment area			Notification interface
**Web-based app**									
	**Directorate General of Health Services**								
		Domiciliary service, health assistant			Ward level: for every 5000-6000 people			Government of Bangladesh–provided tablets (Basail) and project mobile phones (Kasba)	
		**Upazila Health Complex**							
			Resident medical officer	Upazila-facility deaths only			Project mobile phones		
			Nursing supervisor	Upazila-facility deaths in the entire upazila			Project mobile phones		
	**Directorate General of Family Planning**								
		Domiciliary service, family planning assistant (family welfare assistant)			Ward level: for every 5000-6000 people			Government of Bangladesh–provided tablets (Basail) and project mobile phones (Kasba)	
		**Local government body**							
			Union Digital Center, Uddokta	Union level			Project mobile phones		
			Union Parishad, female member	Union level: 25,000 people			Project mobile phones		
**Call center**									
	Local government body, union parishad, village police				Ward level			Call center operator	
	**Self-notification**								
		Family members			Household			Call center operator	
		Imam			Households surrounding the mosques			Call center operator	

### Notification Platform

To facilitate the notification process, we designed 2 platforms: the direct system used an Android smartphone–based app and the indirect one received notifications through a call center. The health assistants, family welfare assistants, resident medical officers, nursing supervisors, members, and Uddoktas notified directly through the app. The village police, imam, and family members notified by calling the call center (Table 1).

To enable notifiers to provide the notifications, the project provided mobile phones preloaded with the app to select notifiers in the Kasba upazila (subdistrict) where the government did not provide tablets for routine data collection among the community health and family planning workers. Project mobile phones were also given to nursing supervisors, female members, and Uddoktas in both upazilas.

### Mobile App

The mobile app was simple and kept to a minimum set of information required for identifying an individual’s household for verification. Our notification system collected information on the identification of an individual birth and death event, date and place of the event, detailed address, and contact. Each entry was stored in the mobile device and the central server. The notification records were made available to the devices of project staff responsible for verification through household visits. The app allowed all notifications to be stored as a new entry, even if there were repeat notifications of the same events (Multimedia Appendix 1).

### Call Center

The call center was based in Dhaka, with 1 operator working between 9 AM and 5 PM. Communication materials were distributed to all households and community informants with the call center number. They left missed calls to the number and they were called back to collect necessary information. For each call, BDT 50 (1 BDT is equivalent to 0.011 USD) was transferred in the form of mobile recharge to families and BDT 20 for imams and village police as incentives. This system also accommodated repeat records from multiple notifiers.

### Household Visit for Verification and Identification of Repeat Notifications

There was no built-in mechanism in the system to flag repeat notifications. It allowed for as many repeat notifications as done by the various notifiers. Each notification was given a unique number generated by the system. We developed a verification system so that we could identify repeat notifications and identify multiple entries for the same individuals. All notifications were verified through confirmation of events by family members during a visit by project staff (a verifier) to the respective household. Verifiers also used a mobile-based platform to collect additional information other than the notifications. They were trained on using the data collection tool and the mobile-based system and given refresher training at regular intervals to ensure quality.

**Figure 2:**
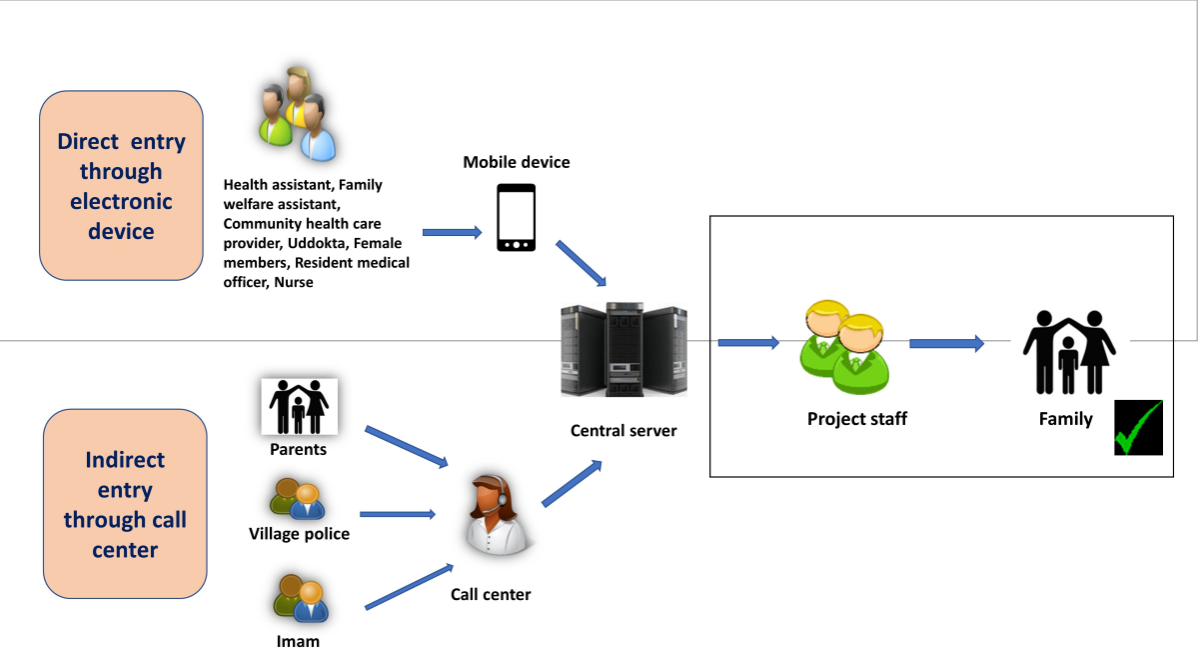
Notification flow and validation.

To understand how the verification system worked, let us assume that 3 different sources notified a single event: a health assistant, the household itself, and an Uddokta. If a notification was first received from the health assistant, our field staff visited the household, collected information to confirm the event and the date and place of the event, and left a calendar at the household with an ID number generated by the system at the first verification visit. This ID number on the calendar helped to identify duplicate notifications during subsequent visits. Assuming that the second notification was received from the household, the field staff went back to the same house and verified the notification. If the event was previously reported by a health assistant, instead of providing a new calendar, he/she would enter the identification number from the existing calendar in the household. The same process would be repeated for the third notification by the Uddokta. Figure 2 entails the notification and verification processes.

### Settings

This project took place in 2 upazilas in Bangladesh: Basail (Tangail district) and Kasba (Brahmanbaria district). We included 5 unions from each upazila for piloting the notification system, covering ~280,000 people from October 2015 to September 2016.

### Assessment of the Functionality of the Notifiers

To assess how well the notification system worked, we conducted a household survey at the end of the notification period between October and December 2016. As this was a pilot, the survey was not designed to measure the accuracy of the different notifiers. Rather, it aimed to assess the coverage/completeness of the notification system that we piloted. Timeliness was measured from the notification system itself through collection of dates of an event during the verification visits. Notifiers could report an event at any time point during our study period. We estimated the time lag between the date of the event and the date of the notification for each notifier. This also helped to identify the notifier who had the shortest time lag between the event and reporting.

For the household survey, we assumed a death rate of 6 per 1000 people and a birth rate of 21 per 1000 people, a recall period of 12 months, and that our notifiers could capture 80% of the birth and death events. An estimated sample of 56,000 people covering 14,000 households was required to assess the notification channels. We defined clusters in the 2 upazilas by using the probability proportionate to size sampling technique. All births and deaths taking place in the surveyed households, accounting for about 20% of total households in the 2 subdistricts between January and October 2016, were recorded. We estimated the number of birth and death events that took place in this random subsample. All households within the selected areas were listed and asked whether any birth or death event took place within the household during the time period mentioned above. If the response was yes, detailed information on the household socioeconomic characteristics, background characteristics of respondents, and the birth and death events was collected. Written consent was obtained from those attending the interview and no monetary compensation was made. There were separate modules for birth and death events. For birth, the women who gave birth were interviewed, while for death, household heads were interviewed. The survey tools are provided in Multimedia Appendices 2 and 3.

### Data Analysis

We used descriptive statistics to elaborate the notification system and the household survey. We entered data into the project database by using Microsoft SQL Server 2005 with Visual Basic 6.0 for the user interface. We used STATA (version 12; StataCorp LLC) for the statistical analysis.

First, we assessed the performance of individual notifiers in terms of proportion of birth/death events notified by each compared to the total unique number of notifications. We also explored coverage reached by the combination of notifiers to identify sources that can reach maximum coverage. The unique notifications were identified based on the calendar ID collected during the repeat verification visits. We also assessed how well each notifier performed in terms of timeliness. Date of event was captured during household verification of each event from household members. Notification coverage was analyzed by notifications received any time after an event and by the first source of notification to understand which source was the quickest in reporting the events.

The next step was to assess the performance of the notifiers compared to the data in the household survey. Using the calendar identification number that was left at the household during the verification visits, we matched the notifications to the information obtained from the household survey. Assuming the survey captured all births and deaths that took place over the study period, we then compared the total births and deaths captured as an overall proportion reported through the notification system to the number reported through the survey. Next, we stratified the birth and death notifications by the source of notification and the place of occurrence of the event.

We compared the demographic characteristics between births and deaths captured and missed by our notification system. We looked at some background characteristics of mothers who gave birth and of household heads who had a deceased member in the family. We separated the analysis by those captured and not captured by our notification channels. We included place of birth, age, sex of child, wealth quintile of household, education, and occupation of mothers who gave birth.

We also tried to investigate any possible geographical pockets where the noncaptured births and deaths took place. We also produced geographical information system–based maps to see whether the events not captured in the notification channels were clustered within any geographic area.

### Ethics Approval

Ethics approval to conduct the study was obtained from the Institutional Review Board of the International Centre for Diarrhoeal Disease Research, Bangladesh (PR 15099).

## Results

### Notifier Performance: Coverage (Proportion of Notifications Done by Each Channel From the Notification Database)

**Figure 3:**
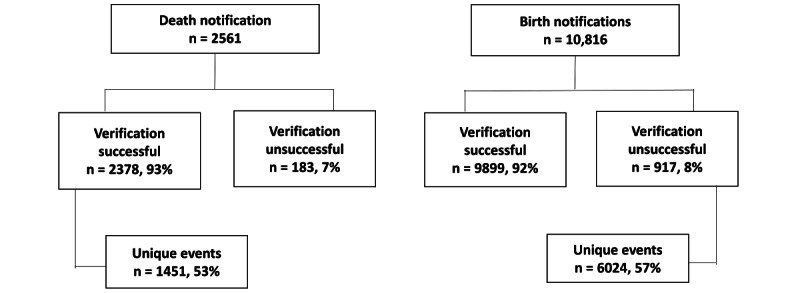
Notification and verification status.

Between January and October 2016, there were 13,377 notifications of births (n=10,816) and deaths (n=2561) received from all channels. All notifications were verified through household visits even in cases of duplicate reports. The verification success rate was 92% for births and 93% for deaths. Health assistants, family welfare assistants, CHCPs, and families were the predominant channels of notification (Figure 3).

Health assistants were the most successful in capturing birth and death events. They covered 76.8% of all births. This was followed by family welfare assistants and CHCPs. Families reported 12.6% of births to the call center. Health assistants were the first to notify 60.4% of the births. Family welfare assistants were the second best performers in terms of notifying births followed by households. A very low proportion of births was notified by facility-level health care providers, namely, nursing supervisors and resident medical officers (Table 2).

Health assistants captured 53.6% (n=778) of all deaths. This was followed by family welfare assistants and CHCPs. Health assistants notified 37.2% (n=540) of deaths before other sources, followed by CHCPs and family welfare assistants. Around 10.1% (n=147) deaths were first notified by family members (Table 2).

**Table 2:** Proportion of births and deaths captured by the individual notification channel in 2 rural subdistricts of Bangladesh from January to October 2016.

Notification processes and channels		Notified anytime		First notifier	
		Birth (N=6024), n (%)	Death (N=1451), n (%)	Birth (N=6024), n (%)	Death (N=1451), n (%)
**Android app**					
	Health assistant	4626 (76.8)	778 (53.6)	3638 (60.4)	540 (37.2)
	Family welfare assistant	1536 (25.5)	402 (27.7)	910 (15.1)	207 (14.3)
	Community health care provider	1012 (16.8)	379 (26.1)	578 (9.6)	254 (17.5)
	Resident medical officer	0 (0)	73 (5)	0 (0)	1 (0.1)
	Nursing supervisor	96 (1.6)	174 (12)	30 (0.5)	4 (0.3)
	Uddokta	48 (0.8)	3 (0.2)	60 (1)	115 (7.9)
	Female member	90 (1.5)	81 (5.6)	78 (1.3)	55 (3.8)
**Call center**					
	Imam	72 (1.2)	68 (4.7)	54 (0.9)	49 (3.4)
	Village police	42 (0.7)	115 (7.9)	30 (0.5)	77 (5.3)
	Parents/family	759 (12.6)	190 (13.1)	645 (10.7)	147 (10.1)

### Notifier Performance: Timeliness

Around 8.4% (n=506) of births were notified on the first day and 71.5% (n=4307) of births were notified within the legally recommended time of 45 days. The median time taken to notify births was 27 (IQR 11-50) days. The median time of notifications via call center was 8 days for births (Table 3). Overall, 31.7% (n=460) of deaths were notified within 1 day while 89.3% (n=1296) were notified within 45 days. The median time taken to notify deaths was 5 (IQR 1-23) days, which was as low as 3 days via the call center (Table 3).

When we consider the timeliness and the source of notification together, median time taken by health assistants to notify births was 34 (IQR 18-55 days). Health assistants notified almost half of these events within 45 days followed by family welfare assistants (Table 3). Median number of days Health assistants took to notify deaths was 9 days and they notified 45% (n=653) of death events within 45 days. Together, health assistants and family welfare assistants notified 55% (n=798) of death events within 45 days (Table 3).

**Table 3:** Notification timeliness by different channels of notification in 2 rural subdistricts of Bangladesh from January to October 2016.

Timeliness			Births (N=6024)		Deaths (N=1451)
**Notification timeliness and source, n (%)**					
	Within 24 hours	506 (8.4)		460 (31.7)	
	Within 72 hours	747 (12.4)		634 (43.7)	
	Within 15 days	1928 (32)		1006 (69.3)	
	Within 45 days	4307 (71.5)		1296 (89.3)	
	Health assistant	2861 (47.6)		653 (45)	
	Family welfare assistant	1145 (19)		353 (24.3)	
**Android-based app, all notifiers (n=168^a^), days, median (IQR)**			27 (11-50)		5 (1-23)
	Health assistant (n=41)	34 (18-55)		9 (3-31)	
	Family welfare assistant (n=49)	17 (6-35)		3 (1-14)	
	Community health care provider (n=34)	27 (11-48)		5 (1-23)	
	Uddokta (n=10)	23 (7- 49)		5 (1-25)	
	Nursing supervisor (n=2)	1 (0-4)		0 (0)	
	Resident medical officer (n=1)	0 (0)		143 (141-146)	
**Call center, days, median (IQR)**			8 (2-33)		3 (1-14)
	Imam	5 (2-12)		1 (0-8)	
	Village police	26 (7-62)		3 (1-17)	
	Parents/family	8 (1-34)		4 (1-14)	

^a^Family members who notified from the community via the call center are not considered among the 168 notifiers.

### Notification Coverage: Findings From Household Survey

The household survey identified 1204 births between January and October 2016. When matched with the notifications, the majority of these births (87.3%, n=1051) were captured by our notification system. Overall, more than half of the births took place in facilities and the rest at home. Among the facility births, a large proportion was notified by community health and family planning workers. The majority (n=367, 65.6%) were notified by health assistants. This was followed by family welfare assistants (n=97, 17.4%), CHCPs (n=86, 15.4%), and households through the call center (n=74, 13.2%). Out of the total 64 deliveries that took place in public facilities, only 2 were notified by the nursing supervisors stationed at the hospitals (n=3, 0.18%) (Table 4). Similarly, among home births, most were notified by health assistants (n=378, 76.8%) followed by family welfare assistants (n=127, 25.8%) and CHCPs (n=69, 14.1%) (Table 4).

**Table 4:** Proportion of births and deaths captured by individual notification channel and place of event in 2 rural subdistricts of Bangladesh from January to October 2016.

Notification process and channels			Household survey										
			Birth (N=1204), n (%)		Home birth (N=547), n (%)		Facility birth (N=657), n (%)		Death (N=341), n (%)		Home death (N=256), n (%)		Facility death (N=85), n (%)
Total			1204 (100)		248 (45.4)		359 (54.6)		341 (100)		192 (75.1)		21 (24.9)
**Android app**													
	Health assistant	743 (70.7)		378 (76.8)		367 (65.6)		109 (49.2)		91 (51.6)		19 (42.4)	
	Family welfare assistant	223 (21.2)		127 (25.8)		97 (17.4)		42 (18.8)		32 (17.9)		10 (21.2)	
	Community health care provider	156 (14.8)		69 (14.1)		86 (15.4)		48 (21.4)		39 (21.9)		9 (20)	
	Resident medical officer	0 (0)		0 (0)		0 (0)		0 (0)		0 (0)		0 (0)	
	Nursing supervisor	4 (0.4)		1 (0.2)		3 (0.5)		1 (0.3)		1 (0.4)		0 (0)	
	Uddokta	11 (1)		3 (0.6)		8 (1.4)		5 (2.4)		3 (1.9)		2 (3.5)	
	Female member	12 (1.1)		3 (0.7)		8 (1.4)		12 (5.6)		10 (5.5)		3 (5.9)	
**Call center**													
	Imam	8 (0.8)		1 (0.2)		7 (1.2)		17 (7.6)		14 (8.2)		3 (5.9)	
	Village police	9 (0.9)		1 (0.2)		8 (1.4)		9 (4.1)		8 (4.7)		1 (2.4)	
	Parents/family	141 (13.4)		66 (13.5)		74 (13.2)		22 (9.7)		16 (8.9)		5 (11.8)	
Total, all sources			1051 (87.3)		492 (89.9)		559 (85.1)		222 (65.1)		176 (68.8)		46 (54.1)

A total of 341 deaths were identified in the household survey. After matching, we found that 65.1% (n=222) of these were captured by our notification system. When analyzed by source of notification, most (n=19, 42.4%) facility deaths were captured by health assistants followed by family welfare assistants. More than half of home deaths were captured by health assistants. This was followed by family welfare assistants (n=32, 17.9%) and CHCPs (n=48, 21.4%) (Table 4).

Among events that were captured both by the notification channels and the survey, 82% (n=4938) of births were notified by health assistants and family welfare assistants combined. Nearly 80% (n=4819) of births were captured by health assistants and CHCPs combined. The proportion increased to 88% (n=5301) for births when the 3 channels were combined. The rest of the sources together could cover around 50% (n=3012) of births and deaths. Nearly 80% (n=1158) of the deaths were captured by health assistants and CHCPs combined. Health assistants, family welfare assistants, and CHCPs together notified 86% (n=1247) of the deaths in the survey. All the remaining sources together covered only around 50% of deaths.

Distribution within the notified and nonnotified births in terms of all these determinants are reported in Table 5. Place of death, sex of deceased, household wealth quintile, education, and occupation of the household head were compared between the notified and nonnotified deaths. A similar distribution pattern was observed in the 2 groups. Statistical significance was not reported owing to the low number of samples in the noncaptured group (Table 5).

**Figure 4:**
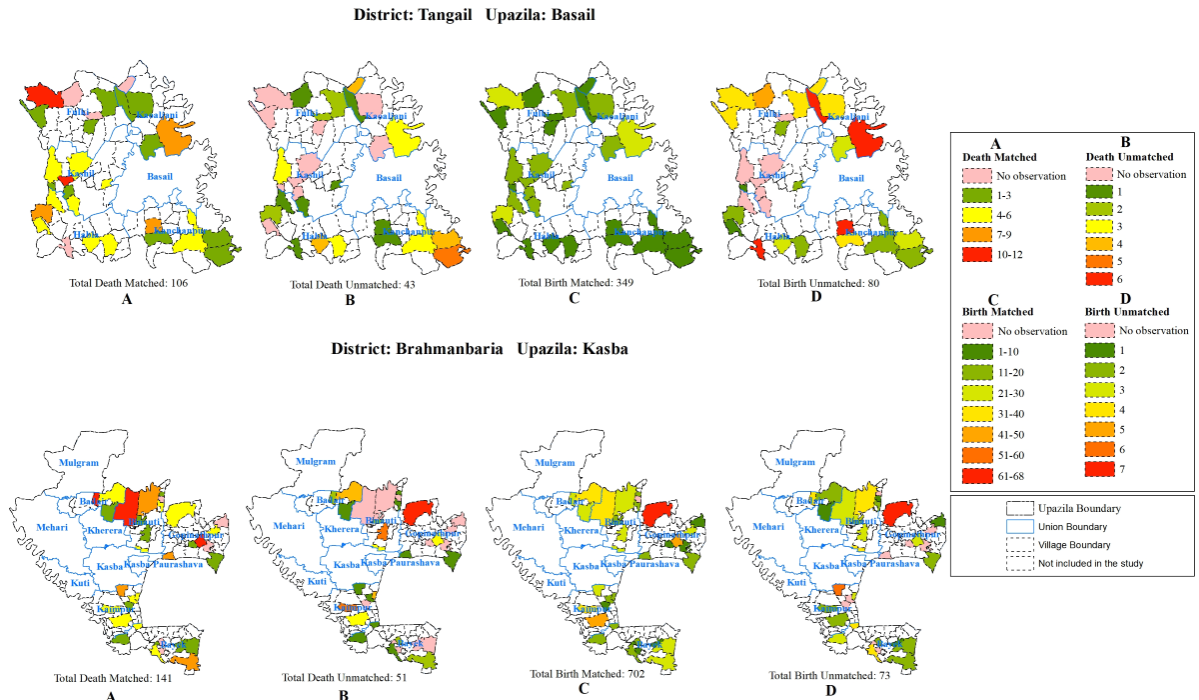
Distribution of births and deaths identified in household surveys across Mouzas (above from left: Basail death notified, death nonnotified, birth notified, birth nonnotified; below from left: Kasba death notified, death nonnotified, birth notified, birth nonnotified).

Our geographical information system maps also suggested no geographic clustering among the nonnotified cases identified during the household survey. Some areas performed better than others, but the untapped birth and death events were evenly scattered around the areas covered by our notifiers (Figure 4).

**Table 5:** Background characteristics of the mothers who gave birth and of decedents in 2 rural subdistricts of Bangladesh from January to October 2016.

		Birth		Death	
		Captured by notification channels (N=1051), n (%)	Not captured by notification channels (N=153), n (%)	Captured by notification channels (N=248), n (%)	Not captured by notification channels (N=94), n (%)
**Place of event**					
	Facility	559 (53.2)	98 (64.1)	54 (21.7)	31 (32.9)
	Home	492 (46.8)	55 (35.9)	194 (78.1)	63 (67)
**Age in years of mother/decedent by cohort**					
	<20	209 (19.9)	39 (25.5)	21 (8.5)	1 (0.8)
	20-29	665 (63.3)	92 (60.1)	37 (14.9)	24 (24.5)
	30-39	166 (15.8)	20 (13.1)	55 (22.3)	24 (25.5)
	40-49	12 (1.1)	2 (1.3)	64 (25.9)	20 (21.3)
	50-59	0 (0)	0 (0)	49 (19.8)	7 (7.5)
	≥60	0 (0)	0 (0)	40 (16.2)	11 (11.7)
	Missing	0 (0)	0 (0)	0 (0)	0 (0)
**Sex**					
	Male	554 (52.7)	86 (56.2)	136 (54.9)	36 (37.8)
	Female	491 (46.7)	66 (43.1)	107 (43.2)	35 (36.9)
	Missing	6 (0.6)	1 (0.7)	5 (1.8)	24 (25.2)
**Wealth quintile**					
	Lowest	186 (17.7)	18 (11.8)	55 (22.1)	21 (22.7)
	Second	197 (18.7)	17 (11.1)	38 (15.3)	19 (20.2)
	Middle	159 (15.1)	38 (24.8)	48 (19.4)	24 (25.2)
	Fourth	167 (15.9)	26 (16.9)	57 (22.9)	13 (14.3)
	Highest	203 (19.3)	28 (18.3)	50 (20.3)	17 (17.7)
**Education (mother/household head)**					
	Primary or below	272 (25.9)	31 (20.3)	235 (94.7)	92 (97.9)
	Secondary	508 (48.3)	68 (44.4)	12 (4.9)	2 (2.1)
	Higher	269 (25.6)	54 (35.3)	1 (0.4)	0 (0)
**Occupation**					
	Unskilled labor	4 (0.4)	0 (0)	27 (10.9)	10 (10.6)
	Skilled worker	34 (3.2)	4 (2.6)	37 (14.9)	18 (19.2)
	Business/trade	3 (0.3)	0 (0)	35 (14.2)	11 (11.7)
	Service holder	3 (0.3)	3 (1.9)	19 (7.7)	3 (3.2)
	Professional	8 (0.8)	0 (0)	5 (2.1)	2 (2.1)
	Unemployed	1000 (95.1)	146 (95.4)	15 (6.1)	2 (2.1)

### Missed Events

Among nonnotified births (n=156), about 35% (n=55) happened outside the project area. One-fifth of the mothers were at their maternal home with newborns. The rest included outmigration (n=41, 26%), false notification (n=16, 10%), and early newborn death (n=9, 6%). Among nonnotified deaths (n=93), approximately 86% (n=80) of the households were not aware of the need for death notification.

## Discussion

### Principal Findings and Policy Recommendations

Our pilot notification system was developed with the aim of identifying a single channel or combination of channels to help improve the notification of birth and death events. Although the notifications from health facilities were very low for both births and deaths, community-level sources, especially the health assistants and family welfare assistants, could together cover more than 80% of the events within their catchment population. Health assistants were the champions in notifying births and deaths with the shortest time lag from the time of the event. Our findings revealed that community-based staff within the routine health system can ensure complete and timely notification of births and deaths [27].

Health assistants and family welfare assistants showcased promising performance in notifying births and deaths within 45 days. Our study identified that coverage can reach as high as 83%, a massive improvement from the 4.5% currently captured in the online birth registration information system [15]. Death notifications have already seen massive improvement as a result of the medical certification of the cause of death and verbal autopsy scale-up initiatives by the government. However, this can also rise drastically from its current state to about 66% through a notification system. We need to keep in mind that the project provided minimal training and facilitation for collecting the notifications. The health sector will be able to achieve even better notification and registration coverage of vital events with proper guidance and regulations from the relevant authorities.

The use of an electronic system and integrating such tools with the current data capture system in Bangladesh can be an effective means to facilitate the notification process. Mobile-based electronic systems for registering births and deaths have been successfully tried out in other countries. In Tanzania, the registrars used a mobile app to collect and upload data to a central system [26]. In Pakistan, marriage contractors, female health workers, and Telenor mobile phone network agents were selected as mobile gatekeepers to reach universal coverage for the national identity scheme [26]. It is also not an additional burden or a completely new system, as Bangladesh itself has done exceptionally well in developing electronic data capture and storage systems. The health assistants and family welfare assistants have already been provided with tablets and training on electronic data capture systems. Once the CRVS++ under the leadership of the cabinet division is in place and running, it will become much easier to accumulate information from the various agents across different ministries, including health, and connect them to the central CRVS database [17].

Poor notifications from facilities emphasize the need to introduce and maintain an accountability mechanism in all public and private facilities for immediate birth and death reporting. Nationally, 37% of births now occur in facilities and 22% of these are in the private sector [27]. Nearly 55% of the deliveries in our survey area took place in facilities. As reported in the household survey, half of these deliveries were conducted in private sector facilities, partly explaining the low level of public facility notification. Although community-level providers captured most of the births and deaths in the study area, health care providers stationed at facilities are much better positioned to provide immediate information on births and deaths. Both private and public facilities are required to report births and deaths in the District Health Information System, which needs a lot of improvements. As an initial step, medical certification of the cause of death forms at both public and private health facilities and verbal autopsy in the community through health assistants have been launched as a pilot to identify the cause of death for generating death certificates [17]. Both these initiatives have played a crucial role in improving death reporting within 45 days.

Although a large proportion of the births still occur at home, frontline domiciliary workers of the government are the best source to inform these events. Domiciliary health and family planning workers have a comparative advantage of “local knowledge” within the community, and notification of births and deaths is part of their day-to-day job. Through regular interaction with households, health assistants and family welfare assistants should be able to quickly and easily learn about important events like births and deaths [28]. This leaves the opportunity for collecting information on births and deaths with a minimum time interval from event occurrence [28].

Our findings indicate some untapped opportunities among CHCPs and Uddoktas in reporting both birth and death events. Fixed duty stations lack delivery facilities, which limits provider-people interactions and is perhaps one of the reasons underlying the low level of reporting by other cadres such as CHCPs or Uddoktas. Although CHCPs work closely with health assistants/family welfare assistants, their work stations and community clinics are a curative platform offering very limited services. Uddoktas, however, are stationed at Union Digital Centers located at the Union Parishad Office, which limits their role to proactively identifying births and deaths. As a demand-side effort, a nationwide campaign on the importance of early registration of births and deaths will increase awareness and accountability within the system [26]. Such mechanisms can be useful for hard-to-reach areas or areas without a dedicated health assistant/family welfare assistant.

The effectiveness of the health sector as a source of birth and death notification, as demonstrated by this study, also opens the door to linking these sources to the local and national level registrar’s office where the registration process will be completed. There are multiple ongoing national initiatives in Bangladesh where digital data systems within the health sector are already underway through introduction of the District Health Information Software and the electronic Management Information System [29,30]. This system can be sustainable, as the Management Information System within the Directorate General of Health Services in Bangladesh is providing handheld tablets to all health assistants. The CHCPs are also equipped with laptop computers. Technical and mechanical difficulties are taken care of by the Management Information System and the Directorate General of Health Services; however, the shortage of adequate monitoring and supervisory bodies as well as technical assistance does slow the digital data input process nationally.

Bangladesh is rich in data in terms of household surveys conducted every 2 or 3 years [24]. Although censuses and household surveys act as a source of vital statistics, these are unable to provide continuous administrative data at the national or subnational level, permitting the production of statistics on population dynamics and health and inequities in service delivery [12]. Moving forward, a complete and functioning notification system based on health sector information relevant to vital events can eventually help replace surveys and censuses. Data collection through surveys and its transition to readable data makes translation of evidence into policy a lengthy process [31]. With declining trends in maternal, newborn, and child mortality, surveys are becoming increasingly resource-intensive, and alternate measures are needed [32,33]. Previous studies suggest that strong CRVS data have been used to estimate maternal mortality, replacing surveys in other settings [34,35].

Simplification of the registration will save time and smoothen the certification process with no hidden fees and can improve registration as seen in a study conducted in Indonesia [23]. Once data are entered in the system, birth and death notifications can be shared in the respective local government offices for processing the birth and death certificates. The notification systems captured all the required information for issuing a certificate. Discussions have already taken place on incorporating all these information fields into the individual tracker in the District Health Information Software-2 and the electronic Management Information System. The only remaining step would be for a family member to physically visit the respective facilities and receive the certificates. Currently, there is no tangible benefit or sense of obligation for parents or individuals for the early registration of births and deaths. Although the government initiated a pilot in Kaligonj subdistrict by using an updated child immunization card to include birth registration numbers, the first vaccine dose is not required until 42 days after birth [36]. This poses the risk of missing early neonatal deaths and stillbirths, which are also commonly missed in household surveys.

Countries with better CRVS systems perform better in terms of their health indicators [10]. Accurate, timely, relevant, comparable, and easily available statistical information is essential for effective program design beyond health [37]. The Vital Statistics Performance Index of Bangladesh is very low [25]. As the country has mandated to strengthen its civil registration system, quick short surveys can eventually be used as a quality check to identify under- or overreporting of births and deaths [7,22]. There is a huge opportunity for capturing deaths through the domiciliary health and family planning workforce. This becomes a much-required and desirable task in a pandemic situation like COVID-19. Our findings suggest that health assistants and family welfare assistants can play a pivotal role in conducting mortality surveillance and support policy makers with the necessary information in mitigation planning [38].

### Limitations

Our study required the verification of every birth/death event by physically visiting each household, which may not be feasible when done nationally. We assessed the innovative digital notification approach by using a sample household survey, whereas conducting a complete census of all households in the study area would be ideal. This was not possible owing to resource constraints, thereby limiting our ability to achieve the objective of matching every birth and death event case by case. This limited our analysis for testing the validity of routine sources and determining an inflation factor for measuring mortality. We also could not include private sector facilities, where a large proportion of facility births take place. However, this was a pilot to test as many sources as possible, and covering a large number of small private clinics was beyond our scope in terms of government-enlisted notifiers. The government in its pilot phase for the identification of cause of death has included private sector facilities. We are also undertaking one new research study to validate the routine health information system in the identification and reporting of adult female mortality. We have included all public and private sector facilities in 2 upazilas. The study, once complete, will add a lot more information to our findings in this paper.

One key limitation for the scale-up of such a program is the lack of information on how to identify duplicates without involving additional human resources. However, much improvement in the national identification card has been made since we conducted our study, and a national identification system will be the key to merging data from various sources and removing duplicates. Another initiative by the government to introduce and implement unique IDs for every individual may facilitate the removal of duplicates in greater capacity as unique IDs include individuals aged 0-18 years as opposed to the national identification card, where the age of eligibility is 18 years.

There are potential sources of bias in reporting births and deaths from the notifiers. We tried addressing that by including as many notifiers as possible from the community. Further, each type of notifier is dedicated to a specific geographical region. To see if there were biases in reporting births and deaths, household characteristics from the survey were compared between notified and missed events. Since a statistical significance test could not be conducted owing to very low numbers, we also looked at the geographic location of the notified and nonnotified events and ensured there was no clustering in reporting. Another source of bias could be the person within the household who was interviewed. There were clear instructions on how to select the respondent (women for birth and household head for death) to minimize such bias.

Finally, this study was undertaken in 2016 and since then, there has been a lot of development in the national CRVS program. The CRVS++ system is under development, and pilot studies to identify the cause of death both from facilities and communities are underway. The pilot study revealed how the routine health information system can be a source of notification for the CRVS system and integration of the two is underway. Through our collaboration with the cabinet division, we have shared our learnings and fed into the pilot that was undertaken in Kaligonj [36]. Our findings will be a very useful addition to evidence-based decision-making for policy makers involved in improving birth and death registration. Apart from improving CRVS, the pilot study also showed ways to improve the measurements and evidence-based decision-making by using the routine health data sources. A larger validation study is ongoing, building onto the pilot study to measure the mortality of female adults from routine sources of information.

### Conclusion

Our pilot study revealed that it is possible to tap into the routine health information system for notification on births and deaths as the first step to ensuring registration. Health assistants captured more than half of the notifications as a stand-alone source and this could be further improved when family welfare assistants are also involved. Timely notification of birth and death events using an innovative communications approach with community-based staff can be a crucial step in improving the registration of births and deaths. Once the notification part is ensured with all the required fields for the certificate made available, the mechanical procedures of certification can easily be completed through the respective local government offices. It can shorten the time needed to issue a certificate and make the process of certification simple and efficient.

## Appendix

Multimedia Appendix 1 Supplementary table.

Multimedia Appendix 2 Census: birth and death module.

Multimedia Appendix 3 Census: death module.

## Abbreviations

CHCP: community health care provider
CRVS: civil registration and vital statistics
