# Supplementary material for: Birth and Death Notifications for Improving Civil Registration and Vital Statistics in Bangladesh: Pilot Exploratory Study
Source: JMIR Public Health Surveill. 2022 Aug 29;8(8):e25735. doi: 10.2196/25735 (PMC9468916; doi:10.2196/25735)
Supplement: Multimedia Appendix 1 [file publichealth_v8i8e25735_app1.docx]

|  |  | **Birth** | | | | | **Death** | | | | |
| --- | --- | --- | --- | --- | --- | --- | --- | --- | --- | --- | --- |
|  | **Mouza** | **Health**  **assistant** | **Community**  **health care**  **provider** | **Family**  **welfare**  **assistant** | **Household** | **Total** | **Health**  **assistant** | **Community**  **health care**  **provider** | **Family**  **welfare**  **assistant** | **Household** | **Total** |
|  | **number** |  |  |  |  |  |  |  |  |  |  |
| n (%) | 22 | 1 (3.33) | 21 (70) | 0 (0) | 6 (20) | 30 (100) | 1 (9.09) | 9 (81.82) | 0 (0) | 4 (36.36) | 11 (100) |
|  |  |  |  |  |  |  |  |  |  |  |  |
| n (%) | 27 | 3 (100) | 0 (0) | 0 (0) | 0 (0) | 3 (100) | 2 (100) | 1 (50) | 0 (0) | 0 (0) | 2 (100) |
|  |  |  |  |  |  |  |  |  |  |  |  |
| n (%) | 55 | 3 (18.75) | 6 (37.5) | 0 (0) | 1 (6.25) | 16 (100) | 1 (33.33) | 1 (33.33) | 0 (0) | 0 (0) | 3 (100) |
|  |  |  |  |  |  |  |  |  |  |  |  |
| n (%) | 66 | 33 (89.19) | 0 (0) | 3 (8.11) | 1 (2.7) | 37 (100) | 5 (55.56) | 0 (0) | 1 (11.11) | 2 (22.22) | 9 (100) |
|  |  |  |  |  |  |  |  |  |  |  |  |
| n (%) | 168 | 19 (67.86) | 0 (0) | 21 (75) | 3 (10.71) | 28 (100) | 4 (57.14) | 0 (0) | 4 (57.14) | 0 (0) | 7 (100) |
|  |  |  |  |  |  |  |  |  |  |  |  |
| n (%) | 178 | 3 (25) | 1 (8.33) | 1 (8.33) | 1 (8.33) | 12 (100) | 0 (0) | 0 (0) | 0 (0) | 0 (0) | 1 (100) |
|  |  |  |  |  |  |  |  |  |  |  |  |
| n (%) | 195 | 12 (66.67) | 0 (0) | 16 (88.89) | 1 (5.56) | 18 (100) | 2 (66.67) | 0 (0) | 2 (66.67) | 0 (0) | 3 (100) |
|  |  |  |  |  |  |  |  |  |  |  |  |
| n (%) | 199 | 18 (75) | 8 (33.33) | 8 (33.33) | 5 (20.83) | 24 (100) | 4 (80) | 3 (60) | 0 (0) | 0 (0) | 5 (100) |
|  |  |  |  |  |  |  |  |  |  |  |  |
| n (%) | 203 | 8 (57.14) | 2 (14.29) | 4 (28.57) | 0 (0) | 14 (100) | 3 (60) | 1 (20) | 0 (0) | 1 (20) | 5 (100) |
|  |  |  |  |  |  |  |  |  |  |  |  |
| n (%) | 258 | 16 (80) | 3 (15) | 8 (40) | 3 (15) | 20 (100) | 0 (0) | 1 (33.33) | 0 (0) | 0 (0) | 3 (100) |
|  |  |  |  |  |  |  |  |  |  |  |  |
| n (%) | 282 | 3 (75) | 0 (0) | 2 (50) | 0 (0) | 4 (100) |  |  |  |  |  |
|  |  |  |  |  |  |  |  |  |  |  |  |
| n (%) | 301 | 18 (85.71) | 6 (28.57) | 1 (4.76) | 5 (23.81) | 21 (100) | 1 (50) | 0 (0) | 0 (0) | 0 (0) | 2 (100) |
|  |  |  |  |  |  |  |  |  |  |  |  |
| n (%) | 313 | 10 (83.33) | 2 (16.67) | 0 (0) | 3 (25) | 12 (100) | 3 (42.86) | 3 (42.86) | 0 (0) | 1 (14.29) | 7 (100) |
|  |  |  |  |  |  |  |  |  |  |  |  |
| n (%) | 344 | 43 (87.76) | 8 (16.33) | 7 (14.29) | 3 (6.12) | 49 (100) | 9 (60) | 6 (40) | 2 (13.33) | 2 (13.33) | 15 (100) |
|  |  |  |  |  |  |  |  |  |  |  |  |
| n (%) | 362 | 14 (66.67) | 1 (4.76) | 15 (71.43) | 2 (9.52) | 21 (100) | 4 (57.14) | 1 (14.29) | 4 (57.14) | 2 (28.57) | 7 (100) |
| n (%) | 380 | 2 (18.18) | 2 (18.18) | 0 (0) | 3 (27.27) | 11 (100) | 0 (0) | 1 (100) | 0 (0) | 0 (0) | 1 (100) |
|  |  |  |  |  |  |  |  |  |  |  |  |
| n (%) | 391 | 5 (23.81) | 3 (14.29) | 0 (0) | 8 (38.1) | 21 (100) | 0 (0) | 0 (0) | 0 (0) | 1 (20) | 5 (100) |
|  |  |  |  |  |  |  |  |  |  |  |  |
| n (%) | 395 | 56 (74.67) | 8 (10.67) | 40 (53.33) | 14 (18.67) | 75 (100) | 6 (50) | 0 (0) | 6 (50) | 0 (0) | 12 (100) |
|  |  |  |  |  |  |  |  |  |  |  |  |
| n (%) | 403 | 17 (68) | 12 (48) | 8 (32) | 2 (8) | 25 (100) | 2 (40) | 2 (40) | 1 (20) | 0 (0) | 5 (100) |
|  |  |  |  |  |  |  |  |  |  |  |  |
| n (%) | 415 | 11 (78.57) | 3 (21.43) | 7 (50) | 1 (7.14) | 14 (100) | 1 (100) | 0 (0) | 0 (0) | 0 (0) | 1 (100) |
|  |  |  |  |  |  |  |  |  |  |  |  |
| n (%) | 424 | 16 (72.73) | 12 (54.55) | 1 (4.55) | 2 (9.09) | 22 (100) | 4 (26.67) | 5 (33.33) | 2 (13.33) | 0 (0) | 15 (100) |
|  |  |  |  |  |  |  |  |  |  |  |  |
| n (%) | 430 | 23 (79.31) | 7 (24.14) | 1 (3.45) | 1 (3.45) | 29 (100) | 7 (70) | 3 (30) | 0 (0) | 1 (10) | 10 (100) |
| n (%) | 434 | 27 (81.82) | 0 (0) | 1 (3.03) | 4 (12.12) | 33 (100) | 12 (85.71) | 0 (0) | 1 (7.14) | 0 (0) | 14 (100) |
|  |  |  |  |  |  |  |  |  |  |  |  |
| n (%) | 458 | 4 (44.44) | 1 (11.11) | 1 (11.11) | 1 (11.11) | 9 (100) | 3 (37.5) | 2 (25) | 0 (0) | 0 (0) | 8 (100) |
|  |  |  |  |  |  |  |  |  |  |  |  |
| n (%) | 489 | 37 (86.05) | 1 (2.33) | 15 (34.88) | 12 (27.91) | 43 (100) | 1 (14.29) | 1 (14.29) | 2 (28.57) | 1 (14.29) | 7 (100) |
|  |  |  |  |  |  |  |  |  |  |  |  |
| n (%) | 491 | 6 (85.71) | 0 (0) | 0 (0) | 0 (0) | 7 (100) | 1 (100) | 0 (0) | 0 (0) | 0 (0) | 1 (100) |
| n (%) | 497 | 16 (84.21) | 0 (0) | 2 (10.53) | 0 (0) | 19 (100) | 2 (25) | 0 (0) | 2 (25) | 0 (0) | 8 (100) |
|  |  |  |  |  |  |  |  |  |  |  |  |
| n (%) | 514 | 5 (23.81) | 3 (14.29) | 0 (0) | 7 (33.33) | 21 (100) | 1 (50) | 0 (0) | 0 (0) | 1 (50) | 2 (100) |
|  |  |  |  |  |  |  |  |  |  |  |  |
| n (%) | 528 | 30 (88.24) | 2 (5.88) | 9 (26.47) | 1 (2.94) | 34 (100) | 3 (33.33) | 1 (11.11) | 2 (22.22) | 2 (22.22) | 9 (100) |
|  |  |  |  |  |  |  |  |  |  |  |  |
| n (%) | 536 | 10 (83.33) | 0 (0) | 0 (0) | 2 (16.67) | 12 (100) | 10 (90.91) | 1 (9.09) | 1 (9.09) | 0 (0) | 11 (100) |
|  |  |  |  |  |  |  |  |  |  |  |  |
| n (%) | 547 | 17 (56.67) | 4 (13.33) | 0 (0) | 6 (20) | 30 (100) | 5 (41.67) | 4 (33.33) | 1 (8.33) | 5 (41.67) | 12 (100) |
|  |  |  |  |  |  |  |  |  |  |  |  |
| n (%) | 552 | 20 (80) | 2 (8) | 6 (24) | 1 (4) | 25 (100) | 6 (66.67) | 2 (22.22) | 5 (55.56) | 0 (0) | 9 (100) |
| n (%) | 581 | 18 (72) | 10 (40) | 0 (0) | 3 (12) | 25 (100) | 6 (66.67) | 4 (44.44) | 0 (0) | 2 (22.22) | 9 (100) |
|  |  |  |  |  |  |  |  |  |  |  |  |
| n (%) | 583 | 13 (76.47) | 2 (11.76) | 15 (88.24) | 0 (0) | 17 (100) | 7 (77.78) | 4 (44.44) | 8 (88.89) | 0 (0) | 9 (100) |
|  |  |  |  |  |  |  |  |  |  |  |  |
| n (%) | 595 | 11 (57.89) | 1 (5.26) | 0 (0) | 3 (15.79) | 19 (100) | 1 (50) | 0 (0) | 0 (0) | 0 (0) | 2 (100) |
|  |  |  |  |  |  |  |  |  |  |  |  |
| n (%) | 603 | 5 (38.46) | 1 (7.69) | 0 (0) | 3 (23.08) | 13 (100) | 0 (0) | 0 (0) | 0 (0) | 0 (0) | 4 (100) |
|  |  |  |  |  |  |  |  |  |  |  |  |
| n (%) | 659 | 9 (60) | 1 (6.67) | 0 (0) | 2 (13.33) | 15 (100) | 0 (0) | 0 (0) | 0 (0) | 1 (25) | 4 (100) |
|  |  |  |  |  |  |  |  |  |  |  |  |
| n (%) | 681 | 27 (87.1) | 3 (9.68) | 1 (3.23) | 3 (9.68) | 31 (100) | 3 (37.5) | 0 (0) | 0 (0) | 0 (0) | 8 (100) |
| n (%) | 715 | 7 (63.64) | 3 (27.27) | 3 (27.27) | 3 (27.27) | 11 (100) | 2 (33.33) | 0 (4) | 0 (2) | 0 (2) | 6 (100) |
|  |  |  |  |  |  |  |  |  |  |  |  |
| n (%) | 721 | 16 (94.12) | 3 (17.65) | 0 (0) | 0 (0) | 17 (100) | 4 (66.67) | 2 (33.33) | 0 (0) | 0 (0) | 6 (100) |
| n (%) | 726 | 15 (78.95) | 1 (5.26) | 0 (0) | 8 (42.11) | 19 (100) | 5 (71.43) | 0 (0) | 0 (0) | 0 (0) | 7 (100) |
|  |  |  |  |  |  |  |  |  |  |  |  |
| n (%) | 736 | 25 (89.29) | 5 (17.86) | 7 (25) | 2 (7.14) | 28 (100) | 1 (33.33) | 2 (66.67) | 2 (66.67) | 0 (0) | 3 (100) |
| n (%) | 749 | 14 (93.33) | 0 (0) | 0 (0) | 1 (6.67) | 15 (100) | 2 (100) | 0 (0) | 0 (0) | 0 (0) | 2 (100) |
|  |  |  |  |  |  |  |  |  |  |  |  |
| n (%) | 838 | 6 (46.15) | 3 (23.08) | 0 (0) | 4 (30.77) | 13 (100) | 1 (20) | 0 (0) | 0 (0) | 0 (0) | 5 (100) |
|  |  |  |  |  |  |  |  |  |  |  |  |
| n (%) | 853 | 24 (100) | 1 (4.17) | 9 (37.5) | 1 (4.17) | 24 (100) | 6 (100) | 2 (33.33) | 4 (66.67) | 0 (0) | 6 (100) |
|  |  |  |  |  |  |  |  |  |  |  |  |
| n (%) | 858 | 78 (77) | 0 (0) | 2 (22.22) | 0 (0) | 9 (100) | 1 (100) | 0 (0) | 1 (100) | 1 (100) | 1 (100) |
|  |  |  |  |  |  |  |  |  |  |  |  |
| n (%) | 871 | 16 (84.21) | 5 (26.32) | 1 (5.26) | 1 (5.26) | 19 (100) | 1 (33.33) | 0 (0) | 0 (0) | 0 (0) | 3 (100) |
|  |  |  |  |  |  |  |  |  |  |  |  |
| n (%) | 881 | 34 (87.18) | 1 (2.56) | 16 (41.03) | 10 (25.64) | 39 (100) | 7 (63.64) | 0 (0) | 7 (63.64) | 0 (0) | 11 (100) |
| n (%) | 916 | 3 (33.33) | 2 (22.22) | 0 (0) | 1 (11.11) | 9 (100) | 1 (16.67) | 0 (0) | 0 (0) | 1 (16.67) | 6 (100) |
|  |  |  |  |  |  |  |  |  |  |  |  |
| n (%) | 924 | 23 (85.19) | 2 (7.41) | 9 (33.33) | 3 (11.11) | 27 (100) | 1 (33.33) | 1 (33.33) | 0 (0) | 0 (0) | 3 (100) |
| n (%) | 927 | 10 (62.5) | 4 (25) | 5 (31.25) | 0 (0) | 16 (100) | 2 (40) | 1 (20) | 2 (40) | 0 (0) | 5 (100) |
| n (%) | 961 | 11 (31.43) | 6 (17.14) | 5 (14.29) | 57 (28) | 35 (100) | 4 (33.33) | 2 (16.67) | 0 (0) | 0 (0) | 12 (100) |
|  |  |  |  |  |  |  |  |  |  |  |  |
| n (%) | 972 | 11 (100) | 0 (0) | 0 (0) | 0 (0) | 11 (100) | 4 (80) | 0 (0) | 0 (0) | 0 (0) | 5 (100) |
| n (%) | 983 | 26 (66.67) | 4 (10.26) | 5 (12.82) | 3 (7.69) | 39 (100) | 4 (40) | 3 (30) | 2 (20) | 0 (0) | 10 (100) |
|  |  |  |  |  |  |  |  |  |  |  |  |
| n (%) | 994 | 14 (100) | 2 (14.29) | 0 (0) | 1 (7.14) | 14 (100) | 2 (50) | 0 (0) | 0 (0) | 3 (75) | 4 (100) |
|  | **Total** | 51  (70.68) | 178  (14.78) | 255  (21.18) | 161  (13.37) | 1204  (100) | 168  (49.27) | 73  (21.41) | 64  (18.77) | 33  (9.68) | 341  (100) |
