# Supplementary material for: Birth and Death Notifications for Improving Civil Registration and Vital Statistics in Bangladesh: Pilot Exploratory Study
Source: JMIR Public Health Surveill. 2022 Aug 29;8(8):e25735. doi: 10.2196/25735 (PMC9468916; doi:10.2196/25735)
Supplement: Multimedia Appendix 2 [file publichealth_v8i8e25735_app2.pdf]

BIRTH & DEATH MODULE (CHILD)

সনাক্তকরণ (Identification)

	নাম Name	কোড Code	
জেলা District		☐☐	সাক্ষাতকার শুরু হবার সময়: Interview starting time: |___|___| : |___|___| ঘন্টা (Hour) মিনিট (Min)
উপজেলা Sub-district		☐☐	
ইউনিয়ন Union		☐☐	
গ্রামের নাম ও কোড Name of village & code		☐☐☐☐☐	
বাড়ির নাম ও নং Name of the house & number		☐☐☐	সাক্ষাতকার শেষ করার সময়: Interview end time: |___|___| : |___|___| ঘন্টা (Hour) মিনিট (Min)
খানা প্রধানের নাম ও খানা নং Name of household head & HH #		☐☐	
June 2015 এরপর জন্মগ্রহণ করা শিশুর মায়ের নাম Name of the mother of the child born after June 2015			
June 2015 এরপর জন্মগ্রহণ করা শিশুর বাবার নাম Name of the father of the child born after June 2015			

সাক্ষাৎকারগ্রহনকারীর পরিদর্শন এবং বর্তমান অবস্থা (Interviewer's visit and status)

	পরিদর্শন-১ Visit 1	পরিদর্শন-২ Visit 2	পরিদর্শন-৩ Visit-3	শেষ পরিদর্শন Final Visit
তারিখ Date	__ __/ __ __/ __ __	__ __/ __ __/ __ __	__ __/ __ __/ __ __	তারিখ Date __ __/ __ __/ __ __
সাক্ষাৎকারগ্রহনকারীর নাম Name of Interviewer				সাক্ষাতকারগ্রহনকারীর কোড (Interviewer code) |___|___|___|
**Result code***	|___|___|	|___|___|	|___|___|	রেজাল্ট কোড* Result code* |___|___|
পরবর্তী পরিদর্শন Next visit	তারিখঃ Date / সময়ঃ Time	তারিখঃ Date / সময়ঃ Time		মোট পরিদর্শন Total # of visit |___|

RESULT CODES*:

01. ইন্টারভিউ সমাপ্ত Interview Complete
02. বাড়ি পরিদর্শনের সময় খানার কোন সদস্যকে বা উপযুক্ত কাউকে পাওয়া যায় নাই No household member or competent respondent were present at home at time of household visit
03. ইন্টারভিউ বাতিল Interview cancelled
04. ইন্টারভিউ দিতে রাজী নয় Refused to give interview
05. বাসস্থানটি খুঁজে পাওয়া যায় নাই Could not find the residence
06. June 2015 এরপর জন্মগ্রহণ করা শিশুর মা অনুপস্থিত mother of the child born after June 2015 is absent
08. অন্যান্য Others ____________________ (উল্লেখ করুন) specify

(Supervision) তত্ত্বাবধায়ন	(Name) নাম	(Code) কোড	(Date) তারিখ
Reviewed by Supervisor		|___|___|	|___|___|-|___|___|-|___|___|
Checked by Field Editor		|___|___|	|___|___|-|___|___|-|___|___|
Reviewed by Office Editor		|___|___|	|___|___|-|___|___|-|___|___|
Keyed by		|___|___|	|___|___|-|___|___|-|___|___|

Informed Consent form

অবহিতকরন সম্মতিপত্র

Protocol No. PR-15099	Version No. 1.00	Date: November, 2016

Protocol Title: বাংলাদেশের টাঙ্গাইল জেলার বাসাইল উপজেলা এবং ব্রাহ্মণবাড়িয়া জেলার কসবা উপজেলায় সিভিল রেজিস্ট্রেশন এর মাধ্যমে শিশু, নবজাতক, মাতৃ এবং অন্যান্য মৃত্যু হার নির্ণয়ের জন্য সময়মত ও পূর্ণ জন্ম এবং মৃত্যু সনাক্তকরণ পদ্ধতি উন্নতিকরন ।

প্রধান তথ্য অনুসন্ধানকারীর নাম **(Principle Investigator's Name):** তাজিন তাহসিনা

গবেষনাকারী প্রতিষ্ঠান **(Organization):** আই সি ডি ডি আর, বি (কলেরা হাসপাতাল)

গবেষণার উদ্দেশ্য (Purpose of the research):

আস্‌সালামুআলাইকুম/ আদাব,

আমরা গবেষণা প্রতিষ্ঠান আই সি ডি ডি আর, বি (কলেরা হাসপাতাল) এ কাজ করি। আমরা আপনার এলাকায় জন্ম ও মৃত্যু সনাক্ত করণের জন্য বিভিন্ন বিকল্প উপায় যাচাই করছি ।ভাইটাল রেজিস্ট্রেশন পদ্ধতি, জাতীয় সিদ্ধান্ত গ্রহনকারীদের, দেশের জনসংখ্যা এবং এর সাথে সম্পর্কিত বিভিন্ন সামাজিক সেবার প্রয়োজনীয়তা সম্পর্কিত তথ্য সরবরাহ করে থাকে। উদাহরন সরূপঃ একটি দেশে কি পরিমান টিকা দরকার, কতগুলো স্বাস্থ্য প্রতিষ্ঠান লাগবে এই জাতীয় । জন্ম নিবন্ধন একজন মানুষের একটি মৌলিক অধিকার এবং এটি একজন শিশুর অস্তিত্ব ও পরিচয় প্রকাশে গুরুত্বপূর্ণ ভূমিকা পালন করে থাকে। এটি শিশুর স্বাস্থ্য ও শিক্ষাগত প্রয়োজনীয়তাও নিশ্চিত করে। অন্যদিকে মৃত্যু নিবন্ধন একটি দেশের অথবা কোনও একটি এলাকার মৃত্যুর পরিমান ও মৃত্যুর কারন অনুমান করতে সাহায্য করে। এবং যেটা কোন দেশের নবজাতক, শিশু এবং প্রাপ্ত বয়স্কদের মৃত্যু হার কমানোর জন্যে আবশ্যকীয় সিদ্ধান্ত গ্রহণে ও পদ্ধতি স্থাপনে খুবই গুরুত্বপূর্ণ ভূমিকা পালন করে।

আপনাকে কেন এই গবেষণায় অন্তর্ভুক্ত করা হলো (Why we have selected you?)

আমরা জন্ম ও মৃত্যু সম্পর্কিত তথ্য সংগ্রহের জন্যে বিদ্যমান বিভিন্ন পন্থা মূল্যায়ন করার এবং প্রকল্পের কেন্দ্রীয় তথ্যভান্ডারে সেগুলো যুক্ত করার পরিকল্পনা গ্রহন করেছি। জন্ম ও মৃত্যু বিষয়ক তথ্য সংগ্রহের সবচেয়ে উত্তম পন্থা অথবা পন্থা সমূহ নির্ণয় করার জন্যে আমাদের একটি নির্দিষ্ট সময়ে এলাকায় সংঘটিত সকল জন্ম ও মৃত্যু সম্পর্কে তথ্য প্রয়োজন। আমরা আমাদের প্রকল্প বাস্তবায়নের শেষের দিকে। আমরা উভয় উপজেলায় সকল খানায় ২০১৬ এর জানুয়ারী থেকে/ এই বছরে ঘটিত সকল জন্ম ও মৃত্যু সংখ্যা রেকর্ড করার জন্যে একটি আদম শুমারি পরিচালনা করছি। এটি আমাদের দুই উপজেলায় নির্দিষ্ট সময়ে ঘটিত সকল জন্ম ও মৃত্যু সংখ্যা সম্পর্কে সঠিক ধারনা দিতে সাহায্য করবে। এরপর আমরা আদম শুমারি থেকে প্রাপ্ত তথ্যের সাথে জন্ম ও মৃত্যু সনাক্তকরণ ও নিবন্ধনের প্রত্যেকটি পদ্ধতি থেকে প্রাপ্ত তথ্যের তুলনা করবো। আপনি যেহেতু এই এলাকার একজন স্থায়ী বাসিন্দা এবং আপনি বলেছেন ২০১৬ এর জানুয়ারী থেকে/ এই বছরে আপনার খানায় জন্ম/ মৃত্যুর ঘটনা ঘটেছে, তাই আমরা আপনাকে এই পাইলট গবেষণার একজন সুবিধাভোগী হিসাবে এই ইন্টারভিউ অংশগ্রহণের আমন্ত্রণ জানাচ্ছি।

কার্যপদ্ধতি ও প্রক্রিয়া (Methods and Procedures)

আমরা আপনাকে আপনার খানা এবং গত এক বছরে আপনার খানায় সংঘটিত জন্ম ও মৃত্যুর নিবন্ধন সম্পর্কে কিছু প্রশ্ন জিজ্ঞাসা করতে চাই। পুরো প্রক্রিয়ায় ২০ থেকে ২৫ মিনিট সময় লাগতে পারে।

ঝুঁকি এবং সুবিধা (Risks and benefits)

এই গবেষণায় অংশগ্রহণের জন্যে আপনার কোন ঝুঁকি নেই । সাক্ষাতকার থেকে প্রাপ্ত সকল তথ্য সম্পূর্ণ গোপন রাখা হবে এবং শুধুমাত্র গবেষণার কাজে ব্যবহার করা হবে। এই গবেষণায় অংশগ্রহণে আপনি সরাসরি উপকৃত নাও হতে পারেন কিন্তু আপনার দেওয়া তথ্যের কার্যকারীতা ব্যাপক। আপনার কাছ থেকে নেয়া তথ্য সমগ্র বাংলাদেশে জনসংখ্যা নিবন্ধনে কার্যকর ভূমিকা রাখবে।

গোপনীয়তা এবং বিশ্বস্ততা (Privacy, anonymity and confidentiality)

আপনাকে নিশ্চয়তা প্রদান করা হচ্ছে যে, আপনাকে চিহ্নিত করার মত সকল তথ্য বিশ্বস্ততার সাথে সম্পূর্ণ গোপন রাখা হবে। আপনি আমাদেরকে যে সকল তথ্য প্রদান করবেন তার সবকিছুই গোপন থাকবে। গবেষণার গবেষক এবং নৈতিক পর্যালোচনা কমিটি (ই আর সি) ছাড়া অন্য কেউই আপনার দেওয়া তথ্য জানতে পারবে না। এমন স্থানে আপনার সাক্ষাৎকার নেয়া হবে যাতে অন্য কেউ কথোপকথন শুনতে না পারে। এই গবেষণা সংক্রান্ত আপনার যে কোন প্রশ্নের উত্তর আমরা সানন্দে দেব।

তথ্যের ভবিষ্যৎ ব্যবহার (Future use of information)
এই গবেষণায় প্রাপ্ত তথ্য পরবর্তীতে দেশে বা দেশের বাইরে অন্য প্রতিষ্ঠানে পুনঃনিরিক্ষণের জন্য পাঠানো হতে পারে। সেক্ষেত্রেও আপনার গোপনীয়তা ও ব্যক্তিগত তথ্য সংরক্ষণ করা হবে যাতে আপনাকে সনাক্ত করা সম্ভব না হয়।

স্বেচ্ছা- সম্মতি (Right not to participate and withdraw)
আপনি এই গবেষণা কার্যক্রমে অংশগ্রহণ করবেন কি করবেন না তা সম্পূর্ণ আপনার স্বতঃস্ফুর্ত সিদ্ধান্ত । আপনি যে প্রশ্নের উত্তর দিতে চান না সেই প্রশ্নের উত্তর দেয়ার কোন প্রয়োজন নেই। অংশগ্রহণ করলেও এই গবেষণা কার্যক্রম চলাকালীন যেকোন সময় আপনি নিজেকে প্রত্যাহার করতে পারবেন। এর জন্য আপনাকে কোন কারণ দর্শাতে হবে না। এমনকি আপনি যদি অংশগ্রহণ নাও করেন সেক্ষেত্রেও আপনার কোনো অসুবিধা হবে না। আমরা আপনার কাছে আবারও আসতে পারি। এমন কোন বাধ্যবাধকতা নাই যে প্রথমবার অংশগ্রহণ করলে পরবর্তীতেও অংশগ্রহণ করতে হবে।

ক্ষতিপূরণের নীতি (Principle of compensation)
আগেই বলা হয়েছে যে এই কাজে আপনার অংশগ্রহণ সম্পূর্ণ স্বতঃস্ফুর্ত এবং এই সাক্ষতকারে অংশগ্রহণের জন্য আপনার কোন সমস্যা বা ক্ষতি হবেনা

যোগাযোগের ঠিকানা (Contact person)
এই প্রকল্পের প্রধান গবেষক তাজিন তাহসিনা, আইসিডিডিআর,বি, মহাখালি, ঢাকা, বাংলাদেশ। এই গবেষণা সর্ম্পকে আপনার যদি কোন প্রশ্ন থাকে তাহলে আপনি তাকে ৮৮১০১১৫ এক্সটেনশন ৩৮০৯ নাম্বারে ফোন করতে পারেন। এবং আপনার যদি অধিকার বা উপকার সর্ম্পকিয় কোন প্রশ্ন থাকে, তবে আপনি জনাব এম এ সালাম খান, কমিটি কোঅর্ডিনেশন সেক্রেটারি, ইথিকস্ রিভিউ কমিটি সেক্রেটারীয়েট, আইসিডিডিআর,বি কে ৯৮৮৬৪৯৮ নাম্বারে যোগাযোগ করতে পারেন ।

আপনি যদি এই গবেষণায় অংশগ্রহণে রাজী থাকেন তাহলে স্বাক্ষর করে সম্মতি দিন। সহযোগিতার জন্য আপনাকে ধন্যবাদ।
অংশগ্রহনকারী: উপরোক্ত তথ্যাবলী আমার কাছে যথাযথভাবে বর্ণিত হয়েছে এবং আমি বর্ণিত তথ্যাবলী বুঝতে পেরে স্ব- ইচ্ছায় এই গবেষণায় অংশগ্রহণে সম্মতি প্রদান করছি।

______________________________ ________________
অংশগ্রহনকারীর স্বাক্ষর তারিখ

স্বাক্ষী: আমি সাক্ষ্য দিচ্ছি যে উপরোক্ত তথ্যাবলী অংশগ্রহনকারীর কাছে যথাযথভাবে বর্ণিত হয়েছে এবং অংশগ্রহনকারী বর্ণিত তথ্যাবলী বুঝে স্ব- ইচ্ছায় এই গবেষণায় অংশগ্রহণে সম্মতি প্রদান করেছে ।

______________________________ ________________
স্বাক্ষীর স্বাক্ষর তারিখ

______________________________ ________________
প্রধান তদন্কারী অথবা তাঁর প্রতিনিধির স্বাক্ষর তারিখ

(উল্লেখ্যঃ প্রধান তদন্তকারীর প্রতিনিধির ক্ষেত্রে তার পূর্ণ নাম ,পদবী এবং সাক্ষর)

Section A: Household Characteristics

এই সেকশনে খানা এবং একই খানার অন্যান্য সদস্যদের কিছু তথ্য সম্বন্ধে আলোচনা করা হয়েছে<br>This section contains some information of the household and other members of the same household				
**No**	**Questions and filters**	**Options and coding category**		**Skip**
A01	আপনার খানায় সাধারণত কতজন লোক বাস করে?<br>How many members usually live in your household?	খানার মোট সদস্য সংখ্যা |____|____| Total # of household members		
A02	আপনার খানায় কতগুলো শোবার ঘর আছে?<br>How many bedrooms do you have in the household?	|____|____|		
A03	আপনার ঘরের/খানার সদস্যদের পানি পান করার প্রধান উৎস কি/কোথায়?<br>What is the main source of drinking water for members of your household?	**পাইপের পানিঃ (Piped water)**		
		বাড়ীর ভিতরে ট্যাপের (পাইপের)পানি (Piped inside dwelling)	11	
		বাড়ীর বাহিরে ট্যাপের (পাইপের) পানি (Piped outside dwelling)	12	
		**কুপের পানিঃ (Well water)**		
		শ্যালো টিউবওয়েল (Shallow tubewell)	21	
		গভীর নলকূপ (Deep tubewell)	22	
		কুয়া (Surface well/other well)	23	
		**ভূ-পৃষ্ঠের পানিঃ (Surface water)**		
		পুকুর/খাল/বদ্ধ জলাশয়/হ্রদ/দীঘি/বিল/হাওড় (Pond/Tank/Lake)	31	
		নদী/ঝর্নার পানি (River Stream)	32	
		বৃষ্টির পানি (Rain water)	41	
		অন্যান্য (Other)<br>(নির্দিষ্ট করুন) (Specify)	97	
A04	আপনাদের খানায় কি ধরণের পায়খানা/ল্যাট্রিন এর ব্যবস্থা আছে?<br>What kind of toilet facility does your household have?	সেপটিক ট্যাংক/আধুনিক ল্যাট্রিন (Septic tank/Modern toilet)	11	
		**গর্ত (পিট) টয়লেট/ল্যাট্রিনঃ** (Pit toilet/Latrine)		
		জলাবদ্ধ/স্ল্যাব (স্যানিটারী) ল্যাট্রিন (Water sealed/Slab latrine)	21	
		গর্তের (পিট) ল্যাট্রিন (Pit latrine)	22	
		খোলা/ঝুলন্ড় ল্যাট্রিন (Open/Hanging latrine)	23	
		ল্যাট্রিন নাই/ঝোপ-ঝাড়/মাঠ (No facility/Bush/Field)	31	
		অন্যান্য Other<br>নির্দিষ্ট করুন (Specify)	97	
A05	আপনাদের ___________ আছে কি, হ্যাঁ হলে, কয়টি আছে?<br>(পশুপাখির নাম)<br>(প্রত্যেকটি পড়ে শোনান)। জানিনা হলে **‘98’** লিখুন, না থাকলে **‘00’** লিখুন।<br>How many of the following animals are owned by your household? Write “98” if don’t know, “00” if none.	গরু Cow .... ☐☐		
		মহিষ Buffalo .... ☐☐		
		ছাগল Goas... ☐☐		
		ভেড়া Sheeps .... ☐☐		
		মুরগী Chicken .... ☐☐		
		হাঁস Ducks... ☐☐		
		কবুতর Pigeons .... ☐☐		

A06	আপনার খানায় বা খানার কোন সদস্যের নিম্নে বর্ণিত জিনিস গুলো আছে কি? Does your household have the following materials?		হ্যাঁ YES	না NO	
		বিদ্যুৎElectricity	1	2	
		রেডিও Radio	1	2	
		টেলিভিশন Television.	1	2	
		মোবাইল ফোন Mobile	1	2	
		টেলিফোন Non-mobile	1	2	
		রেফ্রিজারেটর Refrigerator	1	2	
		আলমারি/ওয়াড্রব Almirah/wardrobe	1	2	
		টেবিল Table	1	2	
		চেয়ার Chair	1	2	
		ইলেকট্রিক পাখা Electric fan	1	2	
		ডিভিডি/ভিসিডি পেল্গয়ার DVD/VCD Player	1	2	
		পানির পাম্প Water pump	1	2	
		টালি Roofing Shingle	1	2	
A07	[পর্যবেক্ষণ করুন] বসত ঘরের দেয়ালের প্রধান নির্মাণ-সামগ্রী *[Observe]* Which material is the wall of the largest structure of the household made of?	**স্বাভাবিক দেয়াল ঃ Natural Walls**			
		দেয়াল নাই No wall		11	
		পাটকাঠি/তাল গাছ/গাছের গুড়ি Cane/Palm/Trunks		12	
		**প্রাথমিক পর্যায়ের দেয়াল Rudimentary Walls**			
		মাটি সহ বাঁশ Bamboo with mud		21	
		মাটিসহ পাথর Stone with mud		22	
		প্লাইউড Plywood		23	
		কার্ডবোর্ড Cardboard		24	
		**পরিপূর্ণ দেয়াল Finished Walls**			
		টিন Tin		31	
		সিমেন্ট Cement		32	
		চুনা পাথর/সিমেন্ট Stone with Lime/Cement		33	
		ইট Bricks		34	
		কাঠের তক্তা Wood Planks/Shingles		35	
		অন্যান্য নির্দিষ্ট করুন Other (Specify)		97	
A08	[পর্যবেক্ষণ করুন] বসত ঘরের মেঝের প্রধান নিমাণ-সামগ্রী *[Observe]* Which material is the floor of the largest room made of?	**স্বাভাবিক মেঝে Natural Floor**			
		মাটি/বালু Earth/Sand		11	
		**প্রাথমিক পর্যায়ের মেঝে Rudimentary Floor**			
		কাঠের তক্তা Wood Planks		21	
		তাল গাছ/বাঁশ Palm/Bamboo		22	
		**পরিপূর্ণ মেঝে Finished Floor**			
		নকশা কাটা কাঠের পাটাতন/পলিশকৃত কাঠ Parquet or Polished Wood		31	
		সিরামিক টাইলস্/মোজাইক Ceramic Tiles		32	
		সিমেন্ট Cement		33	
		অন্যান্য Other		97	
A09	আপনাদের খানার মালিকানায় বসত ভিটা আছে কি? Does your household own any homestead?	হ্যাঁ Yes		1	
		না No		2	

A10	(খানার বসত ভিটা ছাড়া) আপনাদের কোন জমি আছে কি? Does your household own any land (other than the homestead land)?	হ্যাঁ Yes	1	
		না No	2	

Section B: Respondents Background

এই সেকশনে উত্তরদাতা (যে শিশুটি June 2015 এর পর জন্মগ্রহণ করেছে তার মা) সম্পকে কিছু গুরুত্বপূর্ণ তথ্য উলেণ্ডখ করা হয়েছে। This section contains some background information of the respondent (mother of the child that was born after June 2015).
তথ্য সংগ্রহকারি- মা কে বলুন আমি এখন আপনার সম্পর্কে কিছু প্রশ্ন করব।
[For Data Collector- I would like to ask some question about you]

No	Questions and filters	Options and coding category		Skip
B01	আপনার নাম কি?<br>What is your name?	___________________________		
B02	বর্তমানে আপনার বয়স কত?<br>How old were you at your last birthday?	বয়স (পূর্ণ বছরে)<br>Age in completed Years |____|____|		
B03	আপনি কখনও স্কুলে বা মাদ্রাসায় লেখাপড়া করেছেন কি?<br>Has your husband ever attended school/ madrasa?	হ্যাঁ স্কুল Yes School	1	
		হ্যাঁ মাদ্রাসা Yes Madrasa	2	
		হ্যাঁ উভয়ই Yes Both	3	
		না No	4	→B06
B04	আপনি সর্বশেষ কোন্ বিদ্যালয়/স্তর পর্যন্ত পড়াশুনা করেছেন?<br>What is the highest level of school your husband attended: primary, secondary, or higher?	প্রাইমারী/প্রাথমিক Primary	1	
		মাধ্যমিক Secondary	2	
		কলেজ/বিশ্ববিদ্যালয় College/university	3	
B05	আপনি সর্বোচ্চ কোন্ ক্লাশ পাশ করেছেন?<br>What is the highest class your husband completed?	ক্লাশ Class |____|____| (কোন ক্লাশ পাশ না করলে 00 লিখুন।)<br>If completed less than one year, record "00"		
B06	আপনার ধর্ম কি?<br>What is your religion?	মুসলিমMuslim	1	
		হিন্দুHindu.	2	
		খ্রিস্টান Christian	3	
		বৌদ্ধ Buddhist/neo-Buddhist	4	
		অন্যান্য Other<br>(নির্দিষ্ট কর¯ন Specify)	97	

B07	এখন আমি আপনার কাজ সম্বন্ধে কিছু প্রশ্ন করতে চাই। আপনার নিজের ঘর সংসারের কাজ ছাড়া আপনি কি অন্যান্য কিছু করেন যেমন-আপনার ছোট কোন ব্যবসা অথবা পারিবারিক ব্যবসা? Now I would like to ask you some questions about your work. Do you do anything (i.e. have a small business, or work on the family farm or in the family business), apart from your household work?	হ্যাঁYes.	1	
		না No.	2	→B09
B08	আপনার প্রাথমিক পেশা কি, অর্থাৎ আপনি প্রধানত: কি ধরনের কাজ করেন? What is your primary occupation, that is, what kind of work do (did) you mainly do?	**দৈহিক কাজ: Physical work**		
		অদক্ষ কর্মী (যেমন কামলা, মাটি কাটা) Unskilled labour	1	
		দক্ষ কর্মী (যেমন কাঁথা সেলাই, দরজির কাজ, বুটিকের কাজ ইত্যাদি) Skilled worker	2	
		**বিনা কায়িক পরিশ্রম : Non physical work**		
		ব্যবসা Business/trade	3	
		চাকুরীজীবি Service holder	4	
		পেশাজীবি (ডাক্তার/ ইঞ্জিনিয়ার /শিক্ষক) Professional	5	
		অন্যান্যOther (নির্দিষ্ট কর ুন Please specify)	97	
B09	আপনি বর্তমানে বিবাহিতা, বিচ্ছিন্না, পরিত্যক্তা, বিধবা না তালাকপ্রাপ্তা? Are you currently married or separated or deserted or divorced or widowed?	বর্তমানে বিবাহিতা Currently married	1	
		বিচ্ছিন্না Separated	2	→B16
		পরিত্যক্তা Deserted	3	→B16
		তালাকপ্রাপ্তা Divorced	4	→B16
		বিধবা Widowed	5	→B16
B10	বর্তমানে আপনার স্বামীর বয়স কত? How old is he now?	বয়স (পূর্ণ বছরে) Age in completed years |____|____|		
B11	আপনার স্বামী কখনও স্কুলে বা মাদ্রাসায় লেখাপড়া করেছেন কি? Has your husband ever attended school/ madrasa?	হ্যাঁ স্কুল Yes School	1	
		হ্যাঁ মাদ্রাসা Yes Madrasa	2	
		হ্যাঁ উভয়ই Yes Both	3	
		না No	4	→B14
B12	আপনার স্বামী সর্বশেষ কোন্ বিদ্যালয়/স্তর পর্যন্ত পড়াশুনা করেছেন? What is the highest level of school your husband attended: primary, secondary, or higher?	প্রাইমারী/প্রাথমিক Primary	1	
		মাধ্যমিক Secondary	2	
		কলেজ/বিশ্ববিদ্যালয় College/university	3	

B13	আপনার স্বামী সর্বোচ্চ কোন্ ক্লাশ পাশ করেছেন?<br>What is the highest class your husband completed?	ক্লাশ Class |____|____| (কোন ক্লাশ পাশ না করলে 00 লিখুন।)<br>If completed less than one year, record "00"		
B14	বর্তমানে আপনার স্বামী আয় রোজগারের জন্য কোন কাজ করেন কি?<br>Does your husband do anything for living?	হ্যাঁ Yes	1	
		না No	2	→B16
B15	আপনার স্বামীর প্রধান পেশা কি?<br>একাধিক পেশার সাথে জড়িত হলে প্রধান পেশার নাম নিচে লিখে ডান দিকের কোড বৃত্তায়িত করুন।<br>What is his primary occupation, that is, what kind of work does he mainly do?<br>নির্দিষ্টপেশা নীচে লিখুনঃ<br>Write down the occupation below:<br>___________________________________<br>___________________________________	শারীরিক পরিশ্রম ভিত্তিক কাজঃ **Physical work:**		
		নিজের জমিতে চাষাবাদ বা বর্গাচাষী<br>Work on own farm or as a share cropper	1	
		দিন মজুর/অদক্ষ শ্রমিক (গৃহস্থালী,কৃষি ভিত্তিক ইত্যাদি)<br>Day/unskilled laborer (domestic, agricultural and migrant)	2	
		দক্ষ শ্রমিক (কাঠমিস্ত্রী,রাজমিস্ত্রী,জেলে,পাম্পমিস্ত্রী,দোকানে চাকুরী)<br>Skilled worker (carpenter, plumber, shopkeeper)	3	
		রিক্সা চালক/ভ্যান চালক/নৌকা চালক<br>Rickshaw/Van puller/Boat driver	4	
		অ-শারীরিক পরিশ্রম ভিত্তিক কাজঃ **Non physical work:**		
		নিজস্ব ব্যবসা (দোকান/সেলাই/খামার/কুটির শিল্প)<br>Own business	5	
		চাকুরীজীবি/পেশাজীবি (ডাক্তার, প্রকৌশলী, উকিল, শিক্ষক) Service holder/Professionals	6	
		অন্যান্যOther<br>(নির্দিষ্ট করুন Please specify)	97	
B16	আপনার খানায় কত জন উপার্জন ক্ষম সদস্য আছে?<br>How many earning members are there in your household?	|____|____| জন		

SECTION C: Reproductive and birth history:

আপনি June 2015 এরপর যতবার গর্ভবতী হয়েছেন, এবার আমি তার প্রত্যেকটির ব্যাপারে আলোচনা করতে চাই। সেই গর্ভের বা গর্ভাবস্থাগুলো জীবিত শিশু বা মৃতশিশু বা গর্ভ/গর্ভসমূহ মেয়াদের আগে শেষ হয়ে যাওয়া যাই হোক না কেন সবগুলো সম্পকেই আলোচনা করতে চাই। আপনার শেষ গর্ভাবস্থা দিয়েই আলোচনা শুরু করতে চাই। *[যমজ বা একই সাথে হওয়া তার চেয়ে বেশী সংখ্যক সন্তানের ক্ষেত্রে আলাদা লাইন ব্যবহার করুন]*

Now I would like to talk to you about your pregnancies after June 2015, whether the child was born alive, born dead or the pregnancy was lost before full-term, which is as a miscarriage or an abortion. (I would like to start with your last pregnancy. Record Twins and Triplets on separate lines.)

<table>
<tr><th>আপনার শেষ/পূববতী গর্ভাবস্থার কথা চিন্তা করুন</th><th>এই গর্ভে কয়টি বাচ্চা ছিল? এক বা একাধিক (যেমন: যমজ)?
Single/multiple Pregnancy</th><th>কোন বছরের কোন মাসের কত তারিখে এই গর্ভাবস্থা শেষ হয়েছিল?
Date of pregnancy Outcome</th><th>এই গর্ভাবস্থার শিশু কি জীবিত/ মৃতশিশু নাকি মেয়াদের আগে (২৮ সপ্তাহ) নষ্ট হওয়া গভ যেমন: গর্ভনষ্ট বা গর্ভপাত?
Status of pregnancy Outcome</th><th>জন্মের পর বাচ্চাটি কি কেঁদেছিল বা নড়াচড়া করেছিল বা শ্বাসপ্রশ্বাস নিয়েছিল?
Cry/move/breathe after birth</th><th>বাচ্চাটির কি নাম দেয়া হয়েছিল ?
[যদি নাম না থাকে তবে XX লিখুন]
Name of the child</th><th>(নাম) কি মেয়ে না ছেলে?
Sex</th><th>(নাম) কি এখনও জীবিত?
Status of the child (alive or dead)</th><th>যদি জীবিত হয়:
সর্বশেষ বা গত জন্মদিনে (নাম) এর বয়স কত ছিল?
[পূণ বছরের হিসাবে বয়স লিখুন। ১ বছরের কম হলে ০০ লিখূন।]</th><th>যদি মৃত হয়: মৃত্যুর সময় তার বয়স কত ছিল?
[দুই মাস (৬০ দিন) এর কম হলে দিনে, পাঁচ বছরের কম হলে মাসে, পাঁচ বছরের বেশী হলে বছরে লিখুন]</th></tr>
<tr><th>C1</th><th>C2</th><th>C3</th><th>C4</th><th>C5</th><th>C6</th><th>C7</th><th>C8</th><th>C9</th><th>C10</th></tr>
<tr><td>01|</td><td>একক Single 1
একাধিক Multiple... 2
জানি না DK........... 9</td><td>দিন Day………………..|_____|
মাস Month……………|_____|
বছর Year|__|__|__|__|</td><td>জীবিত জন্ম Live birth.............1
(go to C6)
মৃত জন্ম. Still Birth................2
(go to C5)
মেয়াদের আগে নষ্ট Abortion 3
(go to C5)</td><td>হ্যাঁ Yes................. 1
না No.................... 2
(যদি আর কোন গর্ভের ইতিহাস না থাকে তবে SKIP to C11)</td><td>________________
নাম Name</td><td>ছেলে Boy........... 1
মেয়ে Girl........... 2</td><td>হ্যাঁ Yes 1
না No.............. 2
(go to C10)</td><td>বয়স AGE |__|__|
(যদি আর কোন গর্ভের ইতিহাস না থাকে তবে SKIP to C11)</td><td>দিন Day………………..|_____|
মাস Month……………|_____|
বছর Year|__|__|__|__|
(যদি আর কোন গর্ভের ইতিহাস না থাকে তবে skip to C11)</td></tr>
<tr><td>02|</td><td>একক Single 1
একাধিক Multiple... 2
জানি না DK........... 9</td><td>দিন Day………………..|_____|
মাস Month……………|_____|
বছর Year|__|__|__|__|</td><td>জীবিত জন্ম Live birth.............1
(go to C6)
মৃত জন্ম. Still Birth................2
(go to C5)
মেয়াদের আগে নষ্ট Abortion 3
(go to C5)</td><td>হ্যাঁ Yes................. 1
না No.................... 2
(যদি আর কোন গর্ভের ইতিহাস না থাকে তবে SKIP to C11)</td><td>________________
নাম Name</td><td>ছেলে Boy........... 1
মেয়ে Girl........... 2</td><td>হ্যাঁ Yes 1
না No.............. 2
(go to C10)</td><td>বয়স AGE |__|__|
(যদি আর কোন গর্ভের ইতিহাস না থাকে তবে SKIP to C11)</td><td>দিন Day………………..|_____|
মাস Month……………|_____|
বছর Year|__|__|__|__|
(যদি আর কোন গর্ভের ইতিহাস না থাকে তবে skip to C11)</td></tr>
<tr><td>03|</td><td>একক Single 1
একাধিক Multiple... 2
জানি না DK........... 9</td><td>দিন Day………………..|_____|
মাস Month……………|_____|
বছর Year|__|__|__|__|</td><td>জীবিত জন্ম Live birth.............1
(go to C6)
মৃত জন্ম. Still Birth................2
(go to C5)
মেয়াদের আগে নষ্ট Abortion 3
(go to C5)</td><td>হ্যাঁ Yes................. 1
না No.................... 2
(যদি আর কোন গর্ভের ইতিহাস না থাকে তবে SKIP to C11)</td><td>________________
নাম Name</td><td>ছেলে Boy........... 1
মেয়ে Girl........... 2</td><td>হ্যাঁ Yes 1
না No.............. 2
(go to C10)</td><td>বয়স AGE |__|__|
(যদি আর কোন গর্ভের ইতিহাস না থাকে তবে SKIP to C11)</td><td>দিন Day………………..|_____|
মাস Month……………|_____|
বছর Year|__|__|__|__|
(যদি আর কোন গর্ভের ইতিহাস না থাকে তবে skip to C11)</td></tr>
<tr><td>04|</td><td>একক Single 1
একাধিক Multiple... 2
জানি না DK........... 9</td><td>দিন Day………………..|_____|
মাস Month……………|_____|
বছর Year|__|__|__|__|</td><td>জীবিত জন্ম Live birth.............1
(go to C6)
মৃত জন্ম. Still Birth................2
(go to C5)
মেয়াদের আগে নষ্ট Abortion 3
(go to C5)</td><td>হ্যাঁ Yes................. 1
না No.................... 2
(যদি আর কোন গর্ভের ইতিহাস না থাকে তবে SKIP to C11)</td><td>________________
নাম Name</td><td>ছেলে Boy........... 1
মেয়ে Girl........... 2</td><td>হ্যাঁ Yes 1
না No.............. 2
(go to C10)</td><td>বয়স AGE |__|__|
(যদি আর কোন গর্ভের ইতিহাস না থাকে তবে SKIP to C11)</td><td>দিন Day………………..|_____|
মাস Month……………|_____|
বছর Year|__|__|__|__|
(যদি আর কোন গর্ভের ইতিহাস না থাকে তবে skip to C11)</td></tr>
</table>

Note: যদি C8 এর উত্তর না হয়, অর্থাৎ শিশুটি যদি কিছুদিন জীবিত থাকার পরে মারা যায়, তাহলে SECTION D পূরণ শেষে SECTION E পূরণ করুন

যদি C8 এর উত্তর হ্যাঁ হয়, অর্থাৎ শিশুটি যদি জীবিত থাকে , তাহলে SECTION D পর্যন্ত পূরণ করুন

No	Questions and filters	Options and coding category		Skip
C11	শিশুটির জন্ম কোথায় হয়েছে?<br>Where was the child born?	**বাসায় At home :**		
		নিজের বাড়ী Own home	11	
		নানার বাড়ী Maternal grandfather's home	12	
		দাদার বাড়ী Grandfather's home	13	
		অন্য বাড়িতে Other home	14	
		**সরকারি সেবাকেন্দ্র Public sector:**		
		জেলা সদর হাসপাতাল District hospital	21	
		সরকারি মেডিকেল কলেজ হাসপাতাল<br>Government medical college hospital	22	
		মা ও শিশু কল্যাণ কেন্দ্র MCWC	23	
		উপজেলা স্বাস্থ্য কমপেণ্ডক্স UHC	24	
		ইউনিয়ন স্বাস্থ্য ও পরিবার কল্যাণ কেন্দ্র UH&FW	25	
		স্যাটেলাইট ক্লিনিক/ইপি আই সেন্টার<br>Satellite clinic/EPI centre	26	
		কমিউনিটি ক্লিনিক Community clinic	27	
		সরকারি অন্যান্য (উলেণ্টখ করুন)<br>Government Others (Please specify)	28	
		**বেসরকারি সেক্টর NGO sector :**		
		এনজিও স্থায়ী ক্লিনিক/স্বাস্থ্য কেন্দ্র<br>NGO static clinic/health centre	29	
		এনজিও স্যাটেলাইট ক্লিনিক<br>NGO satellite clinic	30	
		এনজিও অন্যান্য (উলেণ্টখ করুন)<br>NGO Others (Please specify)	31	
		**প্রাইভেট মেডিকেল সেক্টর Private medical sector:**		
		প্রাইভেট হাসপাতাল/ ক্লিনিক<br>Pvt.hospital/clinic	32	
		প্রাইভেট মেডিকেল কলেজ হাসপাতাল<br>Private medical college hospital	33	
		প্রাইভেট অন্যান্য (উলেণ্টখ করুন)<br>Private Others (Please specify)	34	

C12	ডেলিভারিটি কে করিয়েছিলেন?<br>Who assisted with the delivery of (NAME)?<br><br>সাক্ষাতকারগ্রহণকারীঃ ডেলিভারিতে প্রধানত যিনি সাহায্য করেছিলেন অর্থ্যাৎ যার ভূমিকা সবচেয়ে বেশি ছিল শুধুমাত্র তার নাম লিখুন এবং তার কোড বৃত্তায়িত করুন।<br><br>নামঃ______________________________<br><br>শুধুমাত্র একটি কোড বৃত্তায়িত করুন।<br><br>Record the name who was mainly responsible or mostly involved in assisting the delivery.<br>Interviewer: Ask and record the name of the person who assisted in delivery<br>Name --<br><br>Circle only one from the list	স্বাস্থ্য পেশাজীবিঃ Health personnel:		
		পাশ করা ডাক্তার MBBS doctor (Qualified)	01	
		নার্স/ ধাত্রী /প্যারামেডিক Nurse/Midwife/Paramedic	02	
		পরিবার কল্যাণ পরিদর্শিকা Family Welfare Visitor (FWV)	03	
		চিকিৎসা সহকারী / উপসহকারী কমিউনিটি চিকিৎসা কর্মকর্তা<br>Medical Assistant / SACMO	04	
		কমিউনিটি ভিত্তিক দক্ষ ধাত্রী<br>Community Skilled Birth Attendant (CSBA)	05	
		কমিউনিটি ক্লিনিক ভিত্তিক স্বাস্থ্য সেবা প্রদানকারী<br>Community Health Care Provider (CHCP)	06	
		স্বাস্থ্য সহকারী Health Assistant (HA)	07	
		পরিবার কল্যাণ সহকারী Family Welfare Assistant (FWA)	08	
		অন্যান্য ব্যক্তিঃ Other person:		
		প্রশিক্ষণপ্রাপ্ত টিবিএ/দাই Trained TBA	09	
		অপ্রশিক্ষিত টিবিএ/ দাই Untrained TBA	10	
		অপ্রশিক্ষিত ডাক্তার (গ্রাম ডাক্তার/ পল্লী চিকিৎসক/ কবিরাজ)<br>Unqualified doctor	11	
		এন জি ও স্বাস্থ্যকর্মী (মা-মনি, ব্রাক, অন্যান্য)<br>NGO health worker (MaMoni/BRAC/Others)	12	
		পরিবারের সদস্য/আত্মীয় Family member / Relative	13	
		প্রতিবেশী/বন্ধু Neighbour / Friend	14	
		অন্যান্য (নির্দিষ্ট করুন)<br>Other: ___________________________________	96	
		জানিনা/মনে নাই Don't know	97	
		কেউ না No one assisted	98	
C13	প্রসবের / ডেলিভারির ধরন কি ছিল?<br>What was the mode of delivery?	স্বাভাবিক প্রসব Normal delivery	1	
		ফরসেপস ডেলিভারি Forceps delivery	2	
		ভ্যাকুয়াম/ ভেনটোস Vacuum/Ventouse	3	
		সিজারিয়ান সেকশন Caesarean section	4	
C14	সাক্ষাতকারগ্রহনকারীঃ প্রশ্ন C11 দেখুন এবং ডেলিভারিটি কোথায় হয়েছে তা বৃত্তায়িত করুন।<br>Check question C11 and circle code of place of delivery.	বাড়িতে At Home (Code 11-14)	1	
		স্বাস্থ্যকেন্দ্রে At Health Facility (Code 21-34)	2	
		অন্যান্য Any other place (Code 96)	3	
C15	সমস্ত সন্তানের মধ্যে (জীবিত, মৃত সহ) বর্তমান শিশু কত নম্বর?<br>What is the serial of the lastborn child among all children (including alive and dead)?	|____|____|		

Section D: Birth Registration

এই সেকশনে খানায় গত June 2015 এরপর থেকে জন্ম নিবন্ধন সম্পর্কে কিছু গুরুত্বপূর্ণ তথ্য উলেণ্ডখ করা হয়েছে। This section contains some information of Birth Registration of the household since June 2015
তথ্য সংগ্রহকারি- মা কে বলুন আমি এখন আপনার খানায় গত এক বছরে জন্ম নিবন্ধন সম্পর্কে কিছু প্রশ্ন করব। যদি একাধিক ব্যাক্তি জন্মগ্রহন করে থাকে তবে সেকশন C এই প্রশ্ন এর সাথে যোগ করুন।] *[For Data Collector- I would like to ask some question about* Birth Registration of your household; *add additional section C with this tool in case of multiple births]*

No	Questions and filters	Options and coding category		Skip
D01	জন্ম ও মৃত্যু নোটিফিকেশন প্রোজেক্ট এর পক্ষ থেকে কোন স্টিকার পেয়েছেন/ পেয়েছিলেন? (কলেরা হাসপাতাল থেকে দেয়া) Did you receive a sticker from birth and death notification project? (Given by icddr,b)	হ্যাঁ Yes	1	
		না No	2	
		জানি না/ মনে নাই Don't know/Can't remember	98	
	স্টিকার টি বাড়ির কোথায় লাগানো, তা পর্যবেক্ষণ করে লিখুন। [স্টিকার লাগানো না থাকলে 99 লিখুন।] Observe where the sticker is and record. [if there is no sticker seen, record 99]	___________________________________		
		লাগানো নাই	99	
D02	এই জন্মের তথ্য কি আপনি বা পরিবারের কেউ কি কলেরা হাসপাতালের দেয়া স্টিকার এর নাম্বার এ ফোন করে জানিয়েছিলেন? Did anyone from the family inform the death to the given number in the sticker provided by icddr,b?	হ্যাঁ Yes	1	→D05
		না No	2	
		জানি না/ মনে নাই Don't know/Can't remember	98	→D05
D03	কেন জানান নি?<br>Why didn't you inform?	প্রকল্পটি সম্পর্কে জানতাম না Didn't know about the project	1	
		প্রয়োজন মনে করিনাই Didn't feel the need to	2	
		হাতের কাছে কোন ফোন ছিলোনা No mobile phone was available	3	
		বাড়ির কারোর মোবাইল ফোন নেই No one in the household owns a mobile phone	4	
		অন্যান্য Other<br>(নির্দিষ্ট করুন Please specify)	98	
D04	শিশুটির জন্মের তথ্য কেউ সংগ্রহ করেছিল কি? Did anyone record the birth of the child?	হ্যাঁ Yes	1	
		না No	2	→D08

D05	জন্ম তথ্য কে সংগ্রহ করেছে? (একাধিক উত্তর হতে পারে) Who did the birth notification of the new born child? (Multiple answer possible)	দক্ষ দাই/ ধাত্রী CSBA	A	
		সেবিকা Nurse	B	
		চিকিৎসক Doctor	C	
		স্বাস্থ্য সহকারী Health Assistant	D	
		পরিবার কল্যান সহকারী FWA	E	
		কমিউনিটি স্বাস্থ্য সেবা প্রদানকারী CHCP	F	
		ইমাম Religious Leader	G	
		গ্রাম চৌকিদার Village Police	H	
		কল সেন্টার Call center	I	
		কলেরা হাসপাতালের পক্ষ থেকে ডাটা কালেক্টর Data collector from icddr,b	J	
		অন্যান্যOther_________________________ (নির্দিষ্ট করুন Please specify)	X	
D06	আপনার খানায় শিশুটির জন্মের তথ্য সংবলিত কোন ক্যালেন্ডার আছে কী? (কলেরা হাসপাতাল থেকে দেয়া) Is there any calendar in your household including birth information of the child? (Given by icddr,b)	হ্যাঁ Yes.	1	
		না No	2	→D08
		জানি না/ মনে নাই Don't know/Can't remember	98	→D08
D07	দয়া করে ক্যালেন্ডারটি দেখান। Please let me see the calendar			
D07A	(প্রশ্নকারী : ক্যালেন্ডারে প্রদত্ত আই ডি নাম্বারটি ভালভাবে দেখে ডান পাশের কলামে লিখুন।) (Observing the calendar, write down the ID No into right side column that is given into calendar.)	ID NO |__|__|__|__|__|__|__|__|__|		
D07B	(প্রশ্নকারী : ক্যালেন্ডারে প্রদত্ত জন্ম তারিখটি ভালভাবে দেখে ডান পাশের কলামে লিখুন।) (Observing the calendar, write down the Date of Birth into right side column that is given in the calendar.)	দিন Day .... |____|____|		
		মাস Month .... |____|____|		
		বছর Year..................... |____|____|____|____|		
D07D	(প্রশ্নকারী : ক্যালেন্ডারে প্রদত্ত জন্ম স্থান ভালভাবে দেখে ডান পাশের কলামে লিখুন।) (Observing the calendar, write down the Place of Birth into right side column that is given in the calendar.)	____		
D08	আপনি বা আপনার পরিবারের কেউ কি ইউনিয়ন পরিষদে গিয়ে শিশুটির জন্ম নিবন্ধন করিয়েছেন? Did you/ anyone in the family do the birth registration of this child?	হ্যাঁ Yes	1	
		না No	2	→END
		জানি না/ মনে নাই Don't know/Can't remember	98	→END
D09	[জন্ম নিবন্ধন সনদ টি দেখতে চান । জন্ম নিবন্ধন সনদ টি দেখে সনদ ইস্যুর তারিখটি লিখুন।] Please record the issue date of the certificate mentioned on it.	দিন Day .... |____|____|		
		মাস Month .... |____|____|		
		বছর Year.....................|____|____|____|____|		

D10	[(প্রশ্নকারী : জন্ম নিবন্ধন সনদ এ প্রদত্ত জন্ম তারিখটি ভালভাবে দেখে ডান পাশের কলামে লিখুন ।)<br>(Observing the birth certificate, write down the Date of Birth into right side column that is given in the calendar.)	দিন Day .... |____|____|		
		মাস Month .... |____|____|		
		বছর Year...................... |____|____|____|____|		
D11	জন্ম নিবন্ধন সনদ টি যে সংগ্রহ করেছে তার সাথে শিশুর সম্পর্ক কি?<br>Who collected the birth certificate?	বাবা Father	1	
		মা Mother	2	
		ভাই Brother	3	
		বোন/ননদ/জা Sister	4	
		চাচা/মামা/খালু/ফুফা Uncle	5	
		চাচী/মামী/খালা/ফুফু Aunt	6	
		দাদী/নানী Grandmother	7	
		দাদা/নানা Grandfather	8	
		অন্যান্য Other: নির্দিষ্ট করুন (specify)____________________	97	

Section E: Death Registration

এই সেকশনে খানায় গত June 2015 এরপর থেকে মৃত্যুনিবন্ধন সম্পর্কে কিছু গুরুত্বপূর্ণ তথ্য উলেলখ করা হয়েছে। This section contains some information of Death Registration of the household since June 2015
[তথ্য সংগ্রহকারি- খানা প্রধানকে বলুন আমি এখন আপনার খানায় গত এক বছরে মৃত্যু নিবন্ধন সম্পর্কে কিছু প্রশ্ন করব। *[For Data Collector- I would like to ask some question about Death Registration of your household]*

No	Questions and filters	Options and coding category		Skip
E01	শিশুটির মৃত্যুর স্থান কোথায় ছিল?<br>What was the place of the death?	বাসায় Home	1	
		স্বাস্থ্যকেন্দ্রে Hospital/ Clinic	2	
		অন্যান্য Other<br>(নির্দিষ্ট করুন Please specify)	97	
E02	শিশুটির কত তারিখে মৃত্যু হয়েছিল?<br>What was the date of the death?	দিন Day |____|____|		
		মাস Month |____|____|		
		বছর Year |____|____|____|____|		
E03	জন্ম ও মৃত্যু নোটিফিকেশন প্রোজেক্ট এর পক্ষ থেকে কোন স্টিকার পেয়েছেন/ পেয়েছিলেন? (কলেরা হাসপাতাল থেকে দেয়া) Did you receive a sticker from birth and death notification project? (Given by icddr,b)	হ্যাঁ Yes	1	
		না No	2	→E4
		জানি না/ মনে নাই Don't know/Can't remember	98	→E4
	স্টিকার টি বাড়ির কোথায় লাগানো, তা পর্যবেক্ষণ করে লিখুন। [স্টিকার লাগানো না থাকলে 99 লিখুন।]<br>Observe where the sticker is and record. [if there is no sticker seen, record 99]	____		
			99	

E04	এই মৃত্যুর তথ্য কি আপনি বা পরিবারের কেউ কি কলেরা হাসপাতালের দেয়া স্টিকার এর নাম্বার এ ফোন করে জানিয়েছিলেন? Did anyone from the family inform the death to the given number in the sticker provided by icddr,b?	হ্যাঁ Yes	1	→C06
		না No	2	
		জানি না/ মনে নাই Don't know/Can't remember	98	→C06
E05	কেন জানান নি? Why didn't you inform?	প্রকল্পটি সম্পর্কে জানতাম না Didn't know about the project	1	
		প্রয়োজন মনে করিনাই Didn't feel the need to	2	
		হাতের কাছে কোন ফোন ছিলোনা Didn't have a mobile phone available	3	
		বাড়ির কারোর মোবাইল ফোন নেই Didn't own a mobile phone	4	
		অন্যান্য Other (নির্দিষ্ট করুন Please specify)	98	
E06	এই মৃত্যুর তথ্য কি কেউ সংগ্রহ করেছিল? Did anybody collect the death information?	হ্যাঁ Yes	1	
		না No	2	→E08
		জানি না/ মনে নাই Don't know/Can't remember	98	→E08
E07	কে সংগ্রহ করেছে ? (একাধিক উত্তর হতে পারে) Who collected information of the dead child? (Multiple answer possible)	সেবিকা Nurse	A	
		চিকিৎসক Doctor	B	
		স্বাস্থ্য সহকারী Health Assistant	C	
		পরিবার কল্যাণ সহকারী FWA	D	
		কমিউনিটি স্বাস্থ্য সেবা প্রদানকারী CHCP	E	
		ইমাম Religious Leader	F	
		গ্রাম চৌকিদার Village Police		
		কল সেন্টার Call center	G	
		কলেরা হাসপাতালের পক্ষ থেকে ডাটা কালেক্টর Data collector from icddr,b	H	
		অন্যান্যOther_________________________ (নির্দিষ্ট করুন Please specify)	X	
E08	আপনার খানায় মৃত্যুর তথ্য সংবলিত কোন ক্যালেন্ডার আছে কী?(কলেরা হাসপাতাল থেকে দেয়া) Is there any calendar in your household including death information of the child who died? (Given by icddr,b)	হ্যাঁ Yes	1	
		না No	2	→E10
		জানি না/ মনে নাই Don't know/Can't remember	98	→E10
E09	দয়া করে ক্যালেন্ডারটি দেখান Please let me see the calendar			
E09a	(ক্যালেন্ডারে প্রদত্ত আই ডি নাম্বারটি ভালভাবে দেখে ডান পাশের কলামে লিখুন।) .(Observing the calendar, interviewer will write down the ID No into right side column that is given in the calendar.)	ID NO: ☐☐☐☐☐☐☐☐☐		

E09b	(প্রশ্নকারী : ক্যালেন্ডারে প্রদত্ত মৃত্যু তারিখটি ভালভাবে দেখে ডান পাশের কলামে লিখুন ।) (Observing the calendar, write down the Date of Death into right side column that is given in the calendar.)	দিন Day .... |____|____|		
		মাস Month .... |____|____|		
		বছর Year..................... |____|____|____|____|		
E09E	(প্রশ্নকারী : ক্যালেন্ডারে প্রদত্ত মৃত্যু স্থান ভালভাবে দেখে ডান পাশের কলামে লিখুন ।) (Observing the calendar, write down the Place of Death into right side column that is given in the calendar.)	______________________________________		
E10	আপনি বা আপনার পরিবারের কেউ কি ইউনিয়ন পরিষদে গিয়ে মৃত শিশুটির মৃত্যু নিবন্ধন করিয়েছেন? Did you/ anyone in the family do the death registration of the deceased?	হ্যাঁ Yes	1	
		না No	2	→END
		জানি না/ মনে নাই Don't know/Can't remember	98	→END
E11	মৃত্যু নিবন্ধন সনদ টি দেখতে চান । মৃত্যু নিবন্ধন সনদ টি দেখে সনদ ইস্যুর তারিখটি লিখুন । Please record the issue date of the certificate mentioned on it.	দিন Day .... |____|____|		
		মাস Month .... |____|____|		
		বছর Year..................... |____|____|____|____|		
E12	[(প্রশ্নকারী : মৃত্যু নিবন্ধন সনদ এ প্রদত্ত মৃত্যু তারিখটি ভালভাবে দেখে ডান পাশের কলামে লিখুন ।) (Observing the death certificate, write down the Date of Death into right side column that is given in the calendar.)	দিন Day .... |____|____|		
		মাস Month .... |____|____|		
		বছর Year......................|____|____|____|____|		
E13	মৃত্যু নিবন্ধন সনদ টি যে সংগ্রহ করেছে তার সাথে মৃত শিশুটির সম্পর্ক কি? Who collected the death certificate?	বাবা Father	1	
		মা Mother	2	
		ভাই Brother	5	
		বোন/ননদ/জা Sister	6	
		চাচা/মামা/খালু/ফুফা Uncle	7	
		চাচী/মামী/খালা/ফুফু Aunt	8	
		দাদী/নানী Grandmother	9	
		দাদা/নানা Grandfather	10	
		অন্যান্য Other: নির্দিষ্ট করুন (specify)_____________________	97	
