# Supplementary material for: Birth and Death Notifications for Improving Civil Registration and Vital Statistics in Bangladesh: Pilot Exploratory Study
Source: JMIR Public Health Surveill. 2022 Aug 29;8(8):e25735. doi: 10.2196/25735 (PMC9468916; doi:10.2196/25735)
Supplement: Multimedia Appendix 3 [file publichealth_v8i8e25735_app3.pdf]

Census: Birth and Death Notification project
International Centre for Diarrhoeal Disease Research, Bangladesh (icddr,b)

DEATH MODULE

সনাক্তকরণ (Identification)

	নাম Name	কোড Code	সাক্ষাতকার শুরু হবার সময়: Interview starting time:
জেলা District		☐☐	|___|___| : |___|___| ঘন্টা (Hour) মিনিট (Min)
উপজেলা Sub-district		☐☐	
ইউনিয়ন Union		☐☐	
গ্রামের নাম ও কোড Name of village & code		☐☐☐☐☐	সাক্ষাতকার শেষ করার সময়: Interview end time:
বাড়ির নাম ও নং Name of the house & number		☐☐☐	
খানা প্রধানের নাম ও খানা নং Name of household head & HH #		☐☐	|___|___| : |___|___| ঘন্টা (Hour) মিনিট (Min)

সাক্ষাৎকারগ্রহনকারীর পরিদর্শন এবং বর্তমান অবস্থা (Interviewer's visit and status)

	পরিদর্শন-১ Visit 1	পরিদর্শন-২ Visit 2	পরিদর্শন-৩ Visit-3
তারিখ Date	__ __/ __ __/ __ __	__ __/ __ __/ __ __	__ __/ __ __/ __ __
সাক্ষাৎকারগ্রহনকারীর নাম Name of Interviewer			
**Result code***	|___|___|	|___|___|	|___|___|
পরবর্তী পরিদর্শন Next visit	তারিখঃ Date<br>সময়ঃ Time	তারিখঃ Date<br>সময়ঃ Time	

শেষ পরিদর্শন Final Visit	
তারিখ Date	__ __/ __ __/ __ __
সাক্ষাতকারগ্রহনকারীর কোড (Interviewer code)	|___|___|___|
রেজাল্ট কোড* Result code*	|___|___|
মোট পরিদর্শন Total # of visit	|___|

RESULT CODES*:

01. ইন্টারভিউ সমাপ্ত Interview Complete
02. বাড়ি পরিদর্শনের সময় খানার কোন সদস্যকে বা উপযুক্ত কাউকে পাওয়া যায় নাই No household member or competent respondent were present at home at time of household visit
03. ইন্টারভিউ বাতিল Interview cancelled
04. ইন্টারভিউ দিতে রাজী নয় Refused to give interview
05. বাসস্থানটি খুঁজে পাওয়া যায় নাই Could not find the residence
06. খানা প্রধান অনুপস্থিত Household Head is absent
08. অন্যান্য Others ________________________ (উল্লেখ করুন) specify

(Supervision) তত্ত্বাবধায়ন	(Name) নাম	(Code) কোড	(Date) তারিখ
Reviewed by Supervisor		|__|__|	|__|__|-|__|__|-|__|__|
Checked by Field Editor		|__|__|	|__|__|-|__|__|-|__|__|
Reviewed by Office Editor		|__|__|	|__|__|-|__|__|-|__|__|
Keyed by		|__|__|	|__|__|-|__|__|-|__|__|

Informed Consent form
অবহিতকরন সম্মতিপত্র

**Protocol No.** PR-**15099**	**Version No. 1.00**	**Date: August 24, 2016**

Protocol Title: বাংলাদেশের টাঙ্গাইল জেলার বাসাইল উপজেলা এবং ব্রাহ্মণবাড়িয়া জেলার কসবা উপজেলায় সিভিল রেজিস্ট্রেশন এর মাধ্যমে শিশু, নবজাতক, মাতৃ এবং অন্যান্য মৃত্যু হার নির্ণয়ের জন্য সময়মত ও পূর্ণ জন্ম এবং মৃত্যু সনাক্তকরণ পদ্ধতি উন্নতিকরন ।

প্রধান তথ্য অনুসন্ধানকারীর নাম **(Principle Investigator's Name):** তাজিন তাহসিনা

গবেষনাকারী প্রতিষ্ঠান **(Organization):** আই সি ডি ডি আর, বি (কলেরা হাসপাতাল)

গবেষণার উদ্দেশ্য (Purpose of the research):

আস্‌সালামুআলাইকুম/ আদাব,

আমরা গবেষণা প্রতিষ্ঠান আই সি ডি ডি আর, বি (কলেরা হাসপাতাল) এ কাজ করি। আমরা আপনার এলাকায় জন্ম ও মৃত্যু সনাক্ত করণের জন্য বিভিন্ন বিকল্প উপায় যাচাই করছি ।ভাইটাল রেজিস্ট্রেশন পদ্ধতি, জাতীয় সিদ্ধান্ত গ্রহনকারীদের, দেশের জনসংখ্যা এবং এর সাথে সম্পর্কিত বিভিন্ন সামাজিক সেবার প্রয়োজনীয়তা সম্পর্কিত তথ্য সরবরাহ করে থাকে। উদাহরন সরূপঃ একটি দেশে কি পরিমান টিকা দরকার, কতগুলো স্বাস্থ্য প্রতিষ্ঠান লাগবে এই জাতীয় । জন্ম নিবন্ধন একজন মানুষের একটি মৌলিক অধিকার এবং এটি একজন শিশুর অস্তিত্ব ও পরিচয় প্রকাশে গুরুত্বপূর্ণ ভূমিকা পালন করে থাকে। এটি শিশুর স্বাস্থ্য ও শিক্ষাগত প্রয়োজনীয়তাও নিশ্চিত করে। অন্যদিকে মৃত্যু নিবন্ধন একটি দেশের অথবা কোনও একটি এলাকার মৃত্যুর পরিমান ও মৃত্যুর কারন অনুমান করতে সাহায্য করে। এবং যেটা কোন দেশের নবজাতক, শিশু এবং প্রাপ্ত বয়স্কদের মৃত্যু হার কমানোর জন্যে আবশ্যকীয় সিদ্ধান্ত গ্রহণে ও পদ্ধতি স্থাপনে খুবই গুরুত্বপূর্ণ ভূমিকা পালন করে।

আপনাকে কেন এই গবেষণায় অন্তর্ভুক্ত করা হলো (Why we have selected you?)

আমরা জন্ম ও মৃত্যু সম্পর্কিত তথ্য সংগ্রহের জন্যে বিদ্যমান বিভিন্ন পন্থা মূল্যায়ন করার এবং প্রকল্পের কেন্দ্রীয় তথ্যভান্ডারে সেগুলো যুক্ত করার পরিকল্পনা গ্রহন করেছি। জন্ম ও মৃত্যু বিষয়ক তথ্য সংগ্রহের সবচেয়ে উত্তম পন্থা অথবা পন্থা সমূহ নির্ণয় করার জন্যে আমাদের একটি নির্দিষ্ট সময়ে এলাকায় সংঘটিত সকল জন্ম ও মৃত্যু সম্পর্কে তথ্য প্রয়োজন। আমরা আমাদের প্রকল্প বাস্তবায়নের শেষের দিকে। আমরা উভয় উপজেলায় সকল খানায় ২০১৬ এর জানুয়ারী থেকে/ এই বছরে ঘটিত সকল জন্ম ও মৃত্যু সংখ্যা রেকর্ড করার জন্যে একটি আদম শুমারি পরিচালনা করছি। এটি আমাদের দুই উপজেলায় নির্দিষ্ট সময়ে ঘটিত সকল জন্ম ও মৃত্যু সংখ্যা সম্পর্কে সঠিক ধারনা দিতে সাহায্য করবে। এরপর আমরা আদম শুমারি থেকে প্রাপ্ত তথ্যের সাথে জন্ম ও মৃত্যু সনাক্তকরণ ও নিবন্ধনের প্রত্যেকটি পদ্ধতি থেকে প্রাপ্ত তথ্যের তুলনা করবো। আপনি যেহেতু এই এলাকার একজন স্থায়ী বাসিন্দা এবং আপনি বলেছেন ২০১৬ এর জানুয়ারী থেকে/ এই বছরে আপনার খানায় জন্ম/ মৃত্যুর ঘটনা ঘটেছে, তাই আমরা আপনাকে এই পাইলট গবেষণার একজন সুবিধাভোগী হিসাবে এই ইন্টারভিউ অংশগ্রহণের আমন্ত্রণ জানাচ্ছি।

কার্যপদ্ধতি ও প্রক্রিয়া (Methods and Procedures)

আমরা আপনাকে আপনার খানা এবং গত এক বছরে আপনার খানায় সংঘটিত জন্ম ও মৃত্যুর নিবন্ধন সম্পর্কে কিছু প্রশ্ন জিজ্ঞাসা করতে চাই। পুরো প্রক্রিয়ায় ২০ থেকে ২৫ মিনিট সময় লাগতে পারে।

ঝুঁকি এবং সুবিধা (Risks and benefits)

এই গবেষণায় অংশগ্রহণের জন্যে আপনার কোন ঝুঁকি নেই । সাক্ষাতকার থেকে প্রাপ্ত সকল তথ্য সম্পূর্ণ গোপন রাখা হবে এবং শুধুমাত্র গবেষণার কাজে ব্যবহার করা হবে। এই গবেষণায় অংশগ্রহণে আপনি সরাসরি উপকৃত নাও হতে পারেন কিন্তু আপনার দেওয়া তথ্যের কার্যকারীতা ব্যাপক। আপনার কাছ থেকে নেয়া তথ্য সমগ্র বাংলাদেশে জনসংখ্যা নিবন্ধনে কার্যকর ভূমিকা রাখবে।

গোপনীয়তা এবং বিশ্বস্ততা (Privacy, anonymity and confidentiality)

আপনাকে নিশ্চয়তা প্রদান করা হচ্ছে যে, আপনাকে চিহ্নিত করার মত সকল তথ্য বিশ্বস্ততার সাথে সম্পূর্ণ গোপন রাখা হবে। আপনি আমাদেরকে যে সকল তথ্য প্রদান করবেন তার সবকিছুই গোপন থাকবে। গবেষণার গবেষক এবং নৈতিক পর্যালোচনা কমিটি (ই আর সি) ছাড়া অন্য কেউই আপনার দেওয়া তথ্য জানতে পারবে না। এমন স্থানে আপনার সাক্ষাৎকার নেয়া হবে যাতে অন্য কেউ কথোপকথন শুনতে না পারে। এই গবেষণা সংক্রান্ত আপনার যে কোন প্রশ্নের উত্তর আমরা সানন্দে দেব।

তথ্যের ভবিষ্যৎ ব্যবহার (Future use of information)

এই গবেষণায় প্রাপ্ত তথ্য পরবর্তীতে দেশে বা দেশের বাইরে অন্য প্রতিষ্ঠানে পুনঃনিরিক্ষণের জন্য পাঠানো হতে পারে। সেক্ষেত্রেও আপনার গোপনীয়তা ও ব্যক্তিগত তথ্য সংরক্ষণ করা হবে যাতে আপনাকে সনাক্ত করা সম্ভব না হয়।

স্বেচ্ছা- সম্মতি (Right not to participate and withdraw)

আপনি এই গবেষণা কার্যক্রমে অংশগ্রহণ করবেন কি করবেন না তা সম্পূর্ণ আপনার স্বতঃস্ফুর্ত সিদ্ধান্ত । আপনি যে প্রশ্নের উত্তর দিতে চান না সেই প্রশ্নের উত্তর দেয়ার কোন প্রয়োজন নেই। অংশগ্রহণ করলেও এই গবেষণা কার্যক্রম চলাকালীন যেকোন সময় আপনি নিজেকে প্রত্যাহার করতে পারবেন। এর জন্য আপনাকে কোন কারণ দর্শাতে হবে না। এমনকি আপনি যদি অংশগ্রহণ নাও করেন সেক্ষেত্রেও আপনার কোনো অসুবিধা হবে না। আমরা আপনার কাছে আবারও আসতে পারি। এমন কোন বাধ্যবাধকতা নাই যে প্রথমবার অংশগ্রহণ করলে পরবর্তীতেও অংশগ্রহণ করতে হবে।

ক্ষতিপূরণের নীতি (Principle of compensation)

আগেই বলা হয়েছে যে এই কাজে আপনার অংশগ্রহণ সম্পূর্ণ স্বতঃস্ফুর্ত এবং এই সাক্ষতকারে অংশগ্রহণের জন্য আপনার কোন সমস্যা বা ক্ষতি হবেনা

যোগাযোগের ঠিকানা (Contact person)

এই প্রকল্পের প্রধান গবেষক তাজিন তাহসিনা, আইসিডিডিআর,বি, মহাখালি, ঢাকা, বাংলাদেশ। এই গবেষণা সর্ম্পকে আপনার যদি কোন প্রশ্ন থাকে তাহলে আপনি তাকে ৮৮১০১১৫ এক্সটেনশন ৩৮০৯ নাম্বারে ফোন করতে পারেন। এবং আপনার যদি অধিকার বা উপকার সর্ম্পকিয় কোন প্রশ্ন থাকে, তবে আপনি জনাব এম এ সালাম খান, কমিটি কোঅর্ডিনেশন সেক্রেটারি, ইথিকস্ রিভিউ কমিটি সেক্রেটারীয়েট, আইসিডিডিআর,বি কে ৯৮৮৬৪৯৮ নাম্বারে যোগাযোগ করতে পারেন ।

আপনি যদি এই গবেষণায় অংশগ্রহণে রাজী থাকেন তাহলে স্বাক্ষর করে সম্মতি দিন। সহযোগিতার জন্য আপনাকে ধন্যবাদ।

অংশগ্রহনকারী: উপরোক্ত তথ্যাবলী আমার কাছে যথাযথভাবে বর্ণিত হয়েছে এবং আমি বর্ণিত তথ্যাবলী বুঝতে পেরে স্ব- ইচ্ছায় এই গবেষণায় অংশগ্রহণে সম্মতি প্রদান করছি।

____________________________ ____________________

অংশগ্রহনকারীর স্বাক্ষর তারিখ

স্বাক্ষী: আমি সাক্ষ্য দিচ্ছি যে উপরোক্ত তথ্যাবলী অংশগ্রহনকারীর কাছে যথাযথভাবে বর্ণিত হয়েছে এবং অংশগ্রহনকারী বর্ণিত তথ্যাবলী বুঝে স্ব- ইচ্ছায় এই গবেষণায় অংশগ্রহণে সম্মতি প্রদান করেছে ।

____________________________ ____________________

স্বাক্ষীর স্বাক্ষর তারিখ

____________________________ ____________________

প্রধান তদন্কারী অথবা তাঁর প্রতিনিধির স্বাক্ষর তারিখ

(উল্লেখ্যঃ প্রধান তদন্তকারীর প্রতিনিধির ক্ষেত্রে তার পূর্ণ নাম ,পদবী এবং সাক্ষর)

Section A: Household Characteristics

এই সেকশনে খানা এবং একই খানার অন্যান্য সদস্যদের কিছু তথ্য সম্বন্ধে আলোচনা করা হয়েছে
This section contains some information of the household and other members of the same household

No	Questions and filters	Options and coding category		Skip
A01	আপনার খানায় সাধারণত কতজন লোক বাস করে? How many members usually live in your household?	খানার মোট সদস্য সংখ্যা |____|____| Total # of household members		
A02	আপনার খানায় কতগুলো শোবার ঘর আছে? How many rooms do you have for sleeping?	|____|____|		
A03	আপনার ঘরের/খানার সদস্যদের পানি পান করার প্রধান উৎস কি/কোথায়? What is the main source of drinking water for members of your household?	**পাইপের পানিঃ (Piped water)**		
		বাড়ীর ভিতরে ট্যাপের (পাইপের)পানি (Piped inside dwelling)	11	
		বাড়ীর বাহিরে ট্যাপের (পাইপের) পানি (Piped outside dwelling)	12	
		**কুপের পানিঃ (Well water)**		
		শ্যালো টিউবওয়েল (Shallow tubewell)	21	
		গভীর নলকূপ (Deep tubewell)	22	
		কুয়া (Surface well/other well)	23	
		**ভূ-পৃষ্ঠের পানিঃ (Surface water)**		
		পুকুর/খাল/বদ্ধ জলাশয়/হ্রদ/দীঘি/বিল/হাওড় (Pond/Tank/Lake)	31	
		নদী/ঝর্নার পানি (River Stream)	32	
		বৃষ্টির পানি (Rain water)	41	
		অন্যান্য (Other) (নির্দিষ্ট করুন)	97	
A04	আপনাদের খানায় কি ধরণের পায়খানা/ল্যাট্রিন এর ব্যবস্থা আছে? What kind of toilet facility does your household have?	সেপটিক ট্যাংক/আধুনিক ল্যাট্রিন (Septic tank/Modern toilet)	11	
		**গর্ত (পিট) টয়লেট/ল্যাট্রিনঃ** (Pit toilet/Latrine)		
		জলাবদ্ধ/স্ল্যাব (স্যানিটারী) ল্যাট্রিন (Water sealed/Slab latrine)	21	
		গর্তের (পিট) ল্যাট্রিন (Pit latrine)	22	
		খোলা/ঝুলন্ড় ল্যাট্রিন (Open/Hanging latrine)	23	
		ল্যাট্রিন নাই/ঝোপ-ঝাড়/মাঠ (No facility/Bush/Field)	31	
		অন্যান্য Other নির্দিষ্ট করুন (Specify)	97	

A05	আপনাদের ___________ আছে কি, হ্যাঁ হলে, কয়টি আছে? (পশুপাখির নাম) (প্রত্যেকটি পড়ে শোনান)। জানিনা হলে **‘98’** লিখুন, না থাকলে **‘00’** লিখুন। How many of the following animals are owned by your household? Write “98” if don’t know, “00” if none.	গরু Cow .... ☐☐			
		মহিষ Buffalo .... ☐☐			
		ছাগল Goas.. ☐☐			
		ভেড়া Sheeps .... ☐☐			
		মুরগী Chicken .... ☐☐			
		হাঁস Ducks... ☐☐			
		কবুতর Pigeons .... ☐☐			
A06	আপনার খানায় বা খানার কোন সদস্যের নিম্নে বর্ণিত জিনিস গুলো আছে কি? Does your household have the following materials?		হ্যাঁ YES	না NO	
		বিদ্যুৎElectricity	1	2	
		রেডিও Radio	1	2	
		টেলিভিশন Television.	1	2	
		মোবাইল ফোন Mobile	1	2	
		টেলিফোন Non-mobile	1	2	
		রেফ্রিজারেটর Refrigerator	1	2	
		আলমারি/ওয়াড্রব Almirah/wardrobe	1	2	
		টেবিল Table	1	2	
		চেয়ার Chair	1	2	
		ইলেকট্রিক পাখা Electric fan	1	2	
		ডিভিডি/ভিসিডি প্লেয়ার DVD/VCD Player	1	2	
		পানির পাম্প Water pump	1	2	
		টালি Roofing Shingle	1	2	
A07	[পর্যবেক্ষণ করুন] বসত ঘরের দেয়ালের প্রধান নির্মাণ-সামগ্রী *[Observe]* Which material is the wall of the largest structure of the household made of?	**স্বাভাবিক দেয়াল ঃ_Natural Walls**			
		দেয়াল নাই No wall	11		
		পাটকাঠি/তাল গাছ/গাছের গুড়ি Cane/Palm/Trunks	12		
		**প্রাথমিক পর্যায়ের দেয়াল Rudimentary Walls**			
		মাটি সহ বাঁশ Bamboo with mud	21		
		মাটিসহ পাথর Stone with mud	22		
		প্লাইউড Plywood	23		
		কার্ডবোর্ড Cardboard	24		

		পরিপূর্ণ দেয়াল **Finished Walls**		
		টিন Tin	31	
		সিমেন্ট Cement	32	
		চুনা পাথর/সিমেন্ট Stone with Lime/Cement	33	
		ইট Bricks	34	
		কাঠের তক্তা Wood Planks/Shingles	35	
		অন্যান্য নির্দিষ্ট করুন<br>Other (Specify)	97	
A08	[পর্যবেক্ষণ করুন] বসত ঘরের মেঝের প্রধান নিমাণ-সামগ্রী<br>*[Observe]* Which material is the floor of the largest room made of?	স্বাভাবিক মেঝে **Natural Floor**		
		মাটি/বালু Earth/Sand	11	
		প্রাথমিক পর্যায়ের মেঝে **Rudimentary Floor**		
		কাঠের তক্তা Wood Planks	21	
		তাল গাছ/বাঁশ Palm/Bamboo	22	
		পরিপূর্ণ মেঝে **Finished Floor**		
		নকশা কাটা কাঠের পাটাতন/পলিশকৃত কাঠ Parquet or Polished Wood	31	
		সিরামিক টাইলস্/মোজাইক Ceramic Tiles	32	
		সিমেন্ট Cement	33	
		অন্যান্য Other<br>(Specify) নির্দিষ্ট করুন	97	
A09	আপনাদের খানার মালিকানায় বসত ভিটা আছে কি? Does your household own any homestead?	হ্যাঁ Yes	1	
		না No	2	
A10	(খানার বসত ভিটা ছাড়া) আপনাদের কোন জমি আছে কি? Does your household own any land (other than the homestead land)?	হ্যাঁ Yes	1	
		না No	2	

Census: Birth and Death Notification project
International Centre for Diarrhoeal Disease Research, Bangladesh (icddr,b)

Section B: Respondents (Household Head) **Background**

এই সেকশনে উত্তরদাতা খানা প্রধান সম্পর্কে কিছু গুরুত্বপূর্ণ তথ্য উলেণ্টখ করা হয়েছে। This section contains some background information of the respondent (houshold head)
তথ্য সংগ্রহকারি- খানা প্রধানকে বলুন আমি এখন আপনার সম্পর্কে কিছু প্রশ্ন করব।
[For Data Collector- I would like to ask some question about you]

No	Questions and filters	Options and coding category		Skip
B01	আপনার নাম কি? What is your name?	___________________________		
B02	বর্তমানে আপনার বয়স কত? How old were you at your last birthday?	বয়স (পূর্ণ বছরে) Age in completed Years |____|____|		
B04	আপনার ধর্ম কি? What is your religion?	মুসলিমMuslim	1	
		হিন্দুHindu.	2	
		খ্রিস্টান Christian	3	
		বৌদ্ধ Buddhist/neo-Buddhist	4	
		অন্যান্য Other (নির্দিষ্ট কর্ন Specify)	97	
B05	আপনি কখনও স্কুলে বা মাদ্রাসায় লেখাপড়া করেছেন কি? Has your husband ever attended school/ madrasa?	হ্যাঁ স্কুল Yes School	1	
		হ্যাঁ মাদ্রাসা Yes Madrasa	2	
		হ্যাঁ উভয়ই Yes Both	3	
		না No	4	→B08
B06	আপনি সর্বশেষ কোন্ বিদ্যালয়/স্তর পর্যন্ত পড়াশুনা করেছেন? What is the highest level of school your husband attended: primary, secondary, or higher?	প্রাইমারী/প্রাথমিক Primary	1	
		মাধ্যমিক Secondary	2	
		কলেজ/বিশ্ববিদ্যালয় College/university	3	
B07	আপনি সর্বোচ্চ কোন্ ক্লাশ পাশ করেছেন? What is the highest class your husband completed?	ক্লাশ Class |____|____| (কোন ক্লাশ পাশ না করলে 00 লিখুন।) If completed less than one year, record "00"		
B08	এখন আমি আপনার কাজ সম্বন্ধে কিছু প্রশ্ন করতে চাই। আপনার নিজের ঘর সংসারের কাজ ছাড়া আপনি কি অন্যান্য কিছু করেন যেমন-আপনার ছোট কোন ব্যবসা অথবা পারিবারিক ব্যবসা? Now I would like to ask you some questions about your work. Do you do anything (i.e. have a small business, or work on the family farm or in the family business), apart from your household work?	হ্যাঁYes.	1	
		না No.	2	→B10

B09	আপনার প্রাথমিক পেশা কি, অর্থাৎ আপনি প্রধানতঃ কি ধরনের কাজ করেন? What is your primary occupation, that is, what kind of work do (did) you mainly do?	**দৈহিক কাজঃ Physical work**		
		অদক্ষ কর্মী (যেমন কামলা, মাটি কাটা) Unskilled labour	1	
		দক্ষ কর্মী (যেমন কাঁথা সেলাই, দরজির কাজ, বুটিকের কাজ ইত্যাদি) Skilled worker	2	
		**বিনা কায়িক পরিশ্রম : Non physical work**		
		ব্যবসা Business/trade	3	
		চাকুরীজীবি Service holder	4	
		পেশাজীবি (ডাক্তার/ ইঞ্জিনিয়ার /শিক্ষক) Professional	5	
		অন্যান্যOther (নির্দিষ্ট করুন Please specify)	97	
B10	আপনার খানায় কত জন উপার্জন ক্ষম সদস্য আছে? How many earning members are there in your household?	|____|____|		

Section C: Death Registration

এই সেকশনে খানায় গত June 2015 মৃত্যুনিবন্ধন সম্পর্কে কিছু গুরুত্বপূর্ণ তথ্য উল্লেখ করা হয়েছে। This section contains some information of Death Registration of the household since June 2015

[তথ্য সংগ্রহকারি- খানা প্রধানকে বলুন আমি এখন আপনার খানায় গত এক বছরে মৃত্যু নিবন্ধন সম্পর্কে কিছু প্রশ্ন করব। যদি একাধিক ব্যাক্তি মৃত্যুবরন করে থাকে তবে সেকশন ডি এই প্রশ্ন এর সাথে যোগ করুন।] *[For Data Collector- I would like to ask some question about Death Registration of your household; add additional section D with this tool in case of multiple deaths]*

No	Questions and filters	Options and coding category		Skip
C01	গত June 2015 থেকে আপনার খানায় কোন সদস্য মৃত্যুবরণ করেছে কি ? In your household, did any member die since June 2015?	হ্যাঁ Yes	1	
		না No	2	→End
C02	মৃত ব্যক্তির নাম কি? What is the name of the dead person?	______________________________		
C03	মৃত ব্যক্তির লিঙ্গ কি? What is the sex of the dead person?	পুরুষ/ Male	1	
		মহিলা/ Female	2	
C04	মৃত্যুর সময় বয়স কত ছিল? What was the age of the dead person during death?	বয়স (পূর্ণ বছরে) Age in completed Years |____|____|		
C05	মৃত্যুর স্থান কোথায় ছিল? What was the place of the death?	বাসায় Home	1	
		স্বাস্থ্যকেন্দ্রে Hospital/ Clinic	2	
		অন্যান্য Other (নির্দিষ্ট করুন Please specify)	97	
C06	কত তারিখে মৃত্যু হয়েছিল? What was the date of the death?	দিন Day |____|____|		
		মাস Month |____|____|		
		বছর Year |____|____|____|____|		

C07	জন্ম ও মৃত্যু নোটিফিকেশন প্রোজেক্ট এর পক্ষ থেকে কোন স্টিকার পেয়েছেন/ পেয়েছিলেন? (কলেরা হাসপাতাল থেকে দেয়া) Did you receive a sticker from birth and death notification project? (Given by icddr,b)	হ্যাঁ Yes	1	
		না No	2	→C8
		জানি না/ মনে নাই Don't know/Can't remember	98	→C8
	স্টিকার টি বাড়ির কোথায় লাগানো, তা পর্যবেক্ষণ করে লিখুন। [স্টিকার লাগানো না থাকলে 99 লিখুন।] Observe where the sticker is and record. [if there is no sticker seen, record 99]	______________________________		
		লাগানো নাই	99	
C08	এই মৃত্যুর তথ্য কি আপনি বা পরিবারের কেউ কি কলেরা হাসপাতালের দেয়া স্টিকার এর নাম্বার এ ফোন করে জানিয়েছিলেন? Did anyone from the family inform the death to the given number in the sticker provided by icddr,b?	হ্যাঁ Yes	1	→C10
		না No	2	
		জানি না/ মনে নাই Don't know/Can't remember	98	→C10
C09	কেন জানান নি? Why didn't you inform?	প্রকল্পটি সম্পর্কে জানতাম না Didn't know about the project	1	
		প্রয়োজন মনে করিনাই Didn't feel the need to	2	
		হাতের কাছে কোন ফোন ছিলোনা Didn't have a mobile phone available	3	
		বাড়ির কারোর মোবাইল ফোন নেই Didn't own a mobile phone	4	
		অন্যান্য Other (নির্দিষ্ট করুন Please specify)	98	
C10	এই মৃত্যুর তথ্য কি কেউ সংগ্রহ করেছিল? Did anybody collect the death information?	হ্যাঁ Yes	1	
		না No	2	→C11
		জানি না/ মনে নাই Don't know/Can't remember	98	→C11
C11	কে সংগ্রহ করেছে ? (একাধিক উত্তর হতে পারে) Who collected information of the dead person? (Multiple answer possible)	সেবিকা Nurse	A	
		চিকিৎসক Doctor	B	
		স্বাস্থ্য সহকারী Health Assistant	C	
		পরিবার কল্যাণ সহকারী FWA	D	
		কমিউনিটি স্বাস্থ্য সেবা প্রদানকারী CHCP	E	
		ইমাম Religious Leader	F	
		গ্রাম চৌকিদার Village Police		
		কল সেন্টার Call center	G	
		কলেরা হাসপাতালের পক্ষ থেকে ডাটা কালেক্টর Data collector from icddr,b	H	
		অন্যান্যOther_________________________ (নির্দিষ্ট করুন Please specify)	X	

C12	আপনার খানায় মৃত্যুর তথ্য সংবলিত কোন ক্যালেন্ডার আছে কী?(কলেরা হাসপাতাল থেকে দেয়া) Is there any calendar in your household including death information of the person who died? (Given by icddr,b)	হ্যাঁ Yes	1	
		না No	2	→C14
		অন্যান্য Other (নির্দিষ্ট করুন Please specify)	97	→C14
C13	দয়া করে ক্যালেন্ডারটি দেখান Please let me see the calendar			
C13a	(ক্যালেন্ডারে প্রদত্ত আই ডি নাম্বারটি ভালভাবে দেখে ডান পাশের কলামে লিখুন।) .(Observing the calendar, interviewer will write down the ID No into right side column that is given in the calendar.)	ID NO: | | | | | | | | | |		
C13b	(প্রশ্নকারী : ক্যালেন্ডারে প্রদত্ত মৃত্যু তারিখটি ভালভাবে দেখে ডান পাশের কলামে লিখুন।) (Observing the calendar, write down the Date of Death into right side column that is given in the calendar.)	দিন Day .... |____|____|		
		মাস Month .... |____|____|		
		বছর Year.................... |____|____|____|____|		
C13c	(প্রশ্নকারী : ক্যালেন্ডারে প্রদত্ত মৃত্যু স্থান ভালভাবে দেখে ডান পাশের কলামে লিখুন।) (Observing the calendar, write down the Place of Death into right side column that is given in the calendar.)	____		
C14	আপনি বা আপনার পরিবারের কেউ কি ইউনিয়ন পরিষদে গিয়ে মৃত ব্যাক্তির মৃত্যু নিবন্ধন করিয়েছেন? Did you/ anyone in the family do the death registration of the deceased?	হ্যাঁ Yes	1	
		না No	2	→END
		জানি না/ মনে নাই Don't know/Can't remember	98	→END
C15	মৃত্যু নিবন্ধন সনদ টি দেখতে চান । মৃত্যু নিবন্ধন সনদ টি দেখে সনদ ইস্যুর তারিখটি লিখুন। Please record the issue date of the certificate mentioned on it.	দিন Day .... |____|____|		
		মাস Month .... |____|____|		
		বছর Year.................... |____|____|____|____|		
C16	[(প্রশ্নকারী : মৃত্যু নিবন্ধন সনদ এ প্রদত্ত মৃত্যু তারিখটি ভালভাবে দেখে ডান পাশের কলামে লিখুন।) (Observing the death certificate, write down the Date of Death into right side column that is given in the calendar.)	দিন Day .... |____|____|		
		মাস Month .... |____|____|		
		বছর Year.....................|____|____|____|____|		
C17	মৃত্যু নিবন্ধন সনদ টি যে সংগ্রহ করেছে তার সাথে মৃত ব্যাক্তির সম্পর্ক কি? Who collected the death certificate?	বাবা Father	1	
		মা Mother	2	
		স্বামী Husband	3	
		স্ত্রী Wife	4	
		ভাই Brother	5	
		বোন/ননদ/জা Sister	6	
		চাচা/মামা/খালু/ফুফা Uncle	7	
		চাচী/মামী/খালা/ফুফু Aunt	8	
		দাদী/নানী Grandmother	9	
		দাদা/নানা Grandfather	10	
		অন্যান্য Other: নির্দিষ্ট করুন (specify)____________________	97	
